# Nuclear and Chloroplast DNA Variation Provides Insights into Population Structure and Multiple Origin of Native Aromatic Rices of Odisha, India

**DOI:** 10.1371/journal.pone.0162268

**Published:** 2016-09-06

**Authors:** Pritesh Sundar Roy, Gundimeda Jwala Narasimha Rao, Sudipta Jena, Rashmita Samal, Ashok Patnaik, Sasank Sekhar Chyau Patnaik, Nitiprasad Namdeorao Jambhulkar, Srigopal Sharma, Trilochan Mohapatra

**Affiliations:** National Rice Research Institute (formerly Central Rice Research Institute), Cuttack, Odisha, India; National Institute of Plant Genome Research, INDIA

## Abstract

A large number of short grain aromatic rice suited to the agro-climatic conditions and local preferences are grown in niche areas of different parts of India and their diversity is evolved over centuries as a result of selection by traditional farmers. Systematic characterization of these specialty rices has not been attempted. An effort was made to characterize 126 aromatic short grain rice landraces, collected from 19 different districts in the State of Odisha, from eastern India. High level of variation for grain quality and agronomic traits among these aromatic rices was observed and genotypes having desirable phenotypic traits like erect flag leaf, thick culm, compact and dense panicles, short plant stature, early duration, superior yield and grain quality traits were identified. A total of 24 SSR markers corresponding to the hyper variable regions of rice chromosomes were used to understand the genetic diversity and to establish the genetic relationship among the aromatic short grain rice landraces at nuclear genome level. SSR analysis of 126 genotypes from Odisha and 10 genotypes from other states revealed 110 alleles with an average of 4.583 and the Nei’s genetic diversity value (*He*) was in the range of 0.034–0.880 revealing two sub-populations SP 1 (membership percentage-27.1%) and SP 2 (72.9%). At the organelle genomic level for the C/A repeats in PS1D sequence of chloroplasts, eight different plastid sub types and 33 haplotypes were detected. The *japonica* (Nipponbare) subtype (6C7A) was detected in 100 genotypes followed by *O*. *rufipogon* (KF428978) subtype (6C6A) in 13 genotypes while *indica* (93–11) sub type (8C8A) was seen in 14 genotypes. The tree constructed based on haplotypes suggests that short grain aromatic landraces might have independent origin of these plastid subtypes. Notably a wide range of diversity was observed among these landraces cultivated in different parts confined to the State of Odisha.

## Introduction

Rice (*Oryza sativa* L.), the staple food crop of world, is also the primary source of living for Asians. Aromatic rice, since known from ancient times, occupied premier position due to consumer preference for their aroma besides other quality traits [1,2]. Among the aromatic rices, long grain Basmatis are highly popular at global level. Apart from these, many indigenous short grain landraces with wide diversity accumulated over thousands of years of natural and artificial selection [3,4] are of preference in their niche areas for speciality preparations. Aromatic short grain rice varieties have been cultivated throughout India with most of them on a small scale in their niche areas, for religious purposes, festivals, entertaining guests and daily use and many of the states have their own distinct set of short grain aromatic rices. The erosion of these valuable resources post-green revolution is of concern from the point of germplasm asset.

Since, rice landraces, the intermediate group between cultivated and wild, are of excellent genetic use for mining of new traits and underlying genes and/or alleles and in view of their importance, systematic characterization of those available in the form of different collections, is of prime importance [5,6]. With the advances in molecular markers, it is now possible to gain valuable insights into the extent of variability at genomic level, thereby hastening identification of duplicates and strengthening their conservation and utilization strategies for future use.

While the cultivation of Basmati rice is restricted to north west India, a specific geographical region, the short grain aromatic rices are spread throughout all Indian states with a high proportion of them are located in several small niche pockets of eastern Indian states. Odisha, comprised of 9 different agro-climatic zones, is one of the major producer and consumer of short grain aromatic rices and possibly have adapted to these varied agro-climatic conditions. In general, the aromatic rices are consumed locally, but have tremendous export potential for their highly acceptable grain and cooking quality traits including strong aroma. However, due to lack of research attention and thorough characterization, many of these excellent genotypes remain untapped. Several of these aromatic rices like Deulabhog, Durgabhog etc form part of integral offerings to Lord Jagannath and Gods of other renowned temples. The Jeypore-Koraput tract of Odisha, recognized as a major biodiversity hot spot and secondary center for origin of rice also houses a rich collection of aromatic rices. A systematic characterization of these native short grain aromatic rices from Odisha is valuable to understand their relation to religion and culture [3].

The present study reports the characterization of native short grain aromatic rice landraces collected from different districts of the Odisha for different agro-morphological and grain quality traits. Further, variability was studied both at both nuclear and chloroplast genome levels so as to establish to understand their origin, inheritance and spread.

## Material and Methods

### Plant material

**Fig 1:**
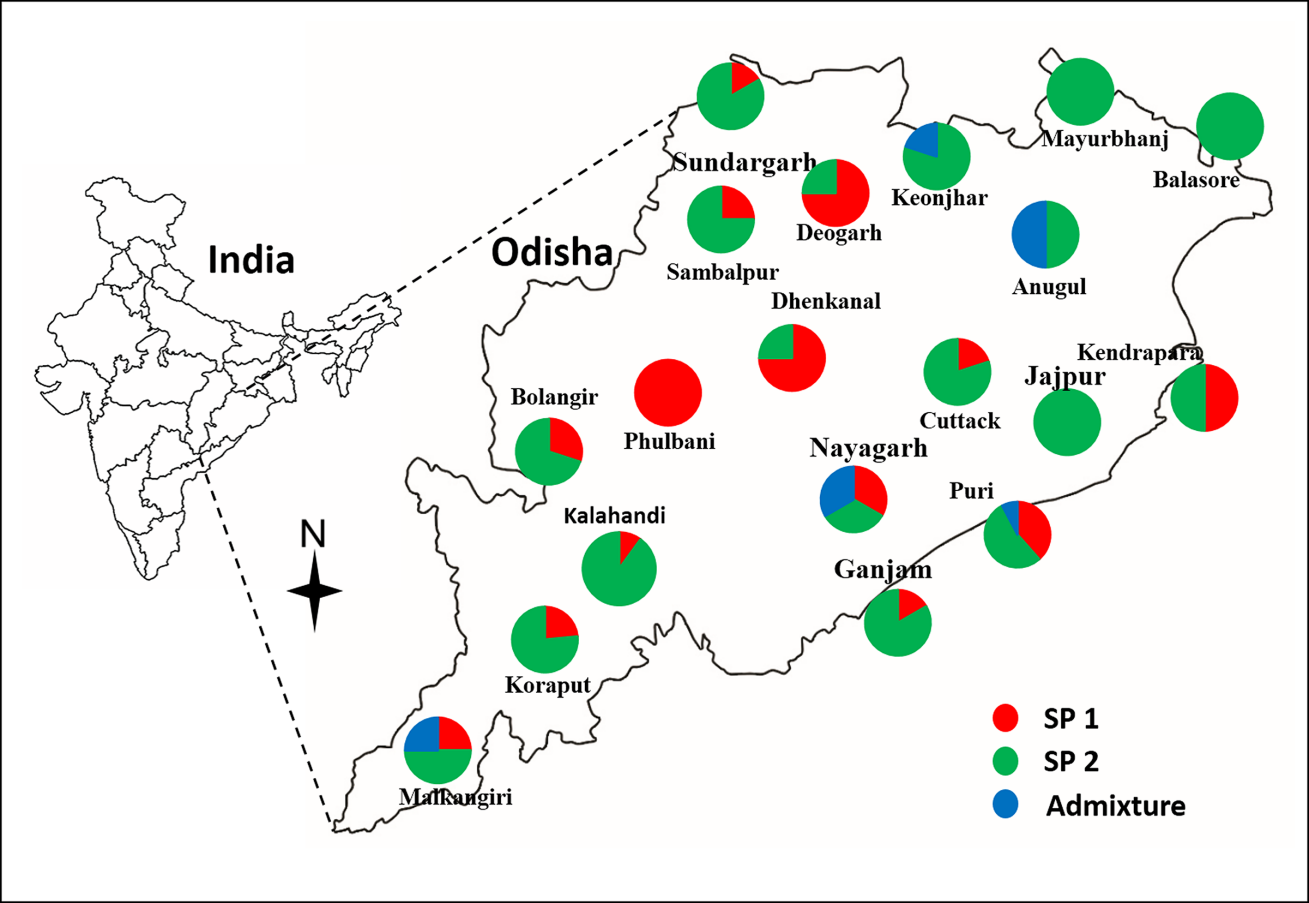
Sampling sites in nineteen districts of Odisha (India) and information on population structure

One hundred and twenty-six short grain aromatic rice lines collected from 19 geographical districts of the State of Odisha, India (Fig 1, S1 Table) were used in this study. The material collections were carried out in the farmers' fields over different locations. Due permission was taken from the respective farmers for the purpose and information regarding passport characters for individual rice landrace, their cultural practices and other necessary information were collected from the farmers with their permission.

### Evaluation of agronomic and grain quality traits

Thirty-day-old seedlings raised from seeds were planted along with 5 released aromatic varieties Nua Kalajeera, Nua Dhusara, Nua Chinikamini, Ketekijoha and Geetanjali as controls with 20 cm × 15 cm spacing in mini-plots of 5 M^2^ following Completely Randomized Block Design with two replications. Standard crop management and protection practices were followed. Data was recorded on agronomic traits like heading duration, plant height, tiller no./plant, and panicle length were recorded from five plants from each genotype. Grain yield was estimated from the produce harvested at maturity from one m^2^ area. Grain quality traits like head rice recovery (HRR), kernel length (KL), kernel breadth (KB), kernel length after cooking (KLAC), volume expansion ratio (VER), water uptake (WU), alkali spreading value (ASV) and amylose content (AC), were determined with grains harvested from the OYT plots. For grain quality, data was collected from five whole milled kernels for estimation of ASV while ten kernels were used for estimation of other traits and all the analysis were performed as per established protocols [7].

### Genotyping using nuclear and chloroplast specific markers

The genomic DNA of rice genotypes was extracted from young green leaves as per Dellaporta *et al*. [8] and the DNA samples were stored at 4°C. For molecular analysis 10 aromatic short grain rice landraces collected from other states (Badshahbhog and Gopalbhog from Madhya Pradesh; Gobindabhog, Sitabhog and Krishnabhog from West Bengal; Dubraj from Chhattisgarh; Katrani from Bihar; Adamchini and two accessions of Kalanamak from Uttar Pradesh) were included in this study to understand the genetic relationship among the aromatic short grain rices of Odisha with those of other states. To assess diversity present in the rice landraces, 24 Rice Microsatellite (RM) markers, two from each chromosome representing all the 12 chromosomes of rice, each amplifying a hyper variable region, were used [9,10]. The chloroplast genome specific marker PD1D, that amplify the conserved plastid sub-types (C and A repeats) of PS1D sequence, was used for classification of rice genotypes based on their wild ancestry [11] (S2 Table).

The PCR reaction mixture contained 50 ng template DNA, 5 picoM of each of forward and reverse primers, 200μM dNTPs, 10X PCR buffer (10 mM Tris-HCl, pH 8.3, 2 mM of MgCl_2_) and 1U Taq (Thermus aquaticus) DNA Polymerase (Genaid, Advanced Molecular Diagnostics, LLC,N, USA). PCR was initially denatured for 5 min at 94°C, then, subjected to 35 cycles of 1min denaturation at 94°C, 1min annealing at 50–61°C (S2 Table) and 1min extension at 72°C; and a final extension for 7min at 72°C and hold at 4°C. The final PCR products were resolved in 2.5% ethidium bromide stained (1μg/ml) agarose gel. The separated PCR products were visualized under UV light and photographed using Typhoon FLA 7000 fluorescent image analyzer (GE Healthcare Bio- Sciences AB, Uppasala, Sweden). For PD1D, the amplified PCR products were directly sequenced. Sequencing reactions were accomplished by the use of Sanger’s dideoxy method of sequencing (www.xcelrislabs.com) in forward direction with 5 μl of each PCR amplification product and the same primers were used for PCR amplification. To compare the maternal inheritance and plastid subtypes of the genotypes, the PD1D primers were blasted in NCBI and compared with sequences of Nipponbare, *O*. *rufipogon* (KF4289781), *O*. *nivara* (AP0067281) and *O*. *indica* (93–11).

### Statistical analysis of data

The test of significance for the agro-morphological data was analyzed by analysis of variance (ANOVA) using the Crop Stat Version 7.2 (International Rice Research Institute, Philippines). Further, Pearson’s correlation coefficients (r) for agronomic characters and grain quality traits were calculated by bivariate procedure using IBM SPSS Statistics (SPSS software, Version 7.5, IBM Corporation, Chicago, USA).

The size of each intense amplified fragment for all SSR loci was determined by comparison with the size standard (100 bp DNA ladder) and scored by incremental numbering from the lowest molecular weight band to the progressively higher molecular weight bands to prepare the genotype matrix. The amplified bands/alleles were scored as present (1) or absent (0) for each genotype and primer combination. The data were entered into a binary matrix and subsequently analyzed using different computer software packages. The polymorphism information content (PIC) for each SSR marker locus was calculated using the formula:$PIC=1-\sum_{j=1}^{n}{\left(Pij\right)}^{2}$, where n is the number of marker alleles for marker i and P_ij_ is the frequency of the j^th^ allele of marker I [12]. Genetic diversity parameters viz., number of alleles (*Na*), effective number of alleles (*Ne*), expected homozygosity (*Ho*), Nei’s genetic diversity index/expected heterozygosity (*He*) [13] and Shannon Index (*I*) were evaluated using POPGENE v 1.32 (http://www.ualberta.ca/fyeh) with 1,000 permutations. The dendrogram based on unbiased genetic distances among genotypes was constructed by un-weighted neighbor joining tree employing MEGA 6 [14]. Principal coordinate analysis (PCoA) was performed based on the simple matching coefficient using the decenter and eigenvector matrices in the software GeneALEx6 [15] with 1,000 random permutations. The Bayesian model-based clustering analysis of the genotypes was used for determining the optimal number of genetic clusters found among rice varieties using the STRUCTURE software [16] with 1,00,000 burn-in periods and 1,00,000 Markov Chain Monte Carlo (MCMC) replicates with ten independent runs (K) ranging from 1 to 10. The ΔK based on the change in the log probability of the data between successive K values were estimated using the parameters described by Evanno *et al*. [17] using the software program Structure Harvester v6.0 [18] and population clusters were produced by the software Structure Plot developed by Ramasamy *et al*. [19] (http://btismysore.in/strplot). Moreover, genotypes were further grouped based on their collection by geographical location and the genetic diversity parameters of the genotypes within each district were determined using POPGENE v 1.32. The genetic variation within and among the populations was calculated by the procedure of AMOVA (Analysis of Molecular Variance) using the software GeneALEx6 [15].

For PD1D, all the sequences were aligned in using Clustal W [20] and manually edited using BioEdit Software version 7.0.9.0 (www.mbio.ncsu.edu). The aligned DNA sequences were imported into DnaSP 5.10 [21] and S (number of polymorphic or segregating sites), p (nucleotide diversity), u (Theta from S, Theta-W), and D (Tajima’s D), nucleotide polymorphism, SNPs and InDels were calculated. Further, the number of haplotypes, haplotype diversity and assignment of genotypes to individual haplotypes were done in DnaSP 5.10 considering gaps/missing. The haplotype tree was constructed with median-joining algorithm and maximum parsimony calculation method using NETWORK 4.6.1.3.

## Results

### Agro-morphological and grain quality trait diversity

A high level of variation for different agronomic and grain quality traits was observed among the genotypes (S3 Table). The plant height ranged from 115.15cm (Dangar Basumati) to 171.95cm (Jayaphul), duration from 115 days (Basanaparijata) to 165 days (Suetpotato) and the yield from 1.57t/ha (Panasmanjee) to 5.52t/ha (Benugopal) (Table 1). Kernel length (KL) ranged from 3.4mm (Dubrajsena) to 7.3mm (Baukunja), kernel breadth (KB) from 1.3mm (Jala, Sujata, Batakarua) to 1.8mm (Bhuinsasal), kernel length after cooking (KLAC) from 6.9mm (Dubrajsena) to 12.3mm (Baukunja), water uptake (WU) from 60 (Acharmati-1) to 158 (Kalazeera), alkali spreading value (ASV) from 2 (Kalikati-1, Atmasital-1, Gangabali) to 7 (Kalazeera) and amylose content (AC) from 16.1% (Basnadhan-1) to 23.1% (Ganjeikali). However, no significant variation was observed for volume expansion ratio (VER).

**Table 1:** Variability for different agronomic and grain quality traits in short grain aromatic rices.

Trait	Mean	Min.	Max.	LSD (5%)	CV (5%)
**Agronomic traits**					
Plant height (cm)	141.12	115.15	171.95	2.06	2.10
Duration (days)	131	115	161	2.93	3.20
Panicle length (cm)	27.42	20.30	33.20	1.39	7.20
EBT	7.57	3.90	12.50	0.51	9.60
Yield (t/ha)	3.23	1.57	5.52	27.14	11.90
**Grain quality traits**					
KL (mm)	4.4	3.4	7.3	-	-
KB (mm)	1.5	1.3	1.8	-	-
KLAC (mm)	8.6	6.9	12.3	-	-
VER	3.7	3.5	4.0	-	-
WU	111	60	158	-	-
ASV	4	2	7	-	-
AC (%)	20.4	16.1	23.1	-	-

EBT: Ear Bearing Tiller; KL: Kernel Length; KB: Kernel Breadth; KLAC: Kernel Length After Cooking; VER: Volume Expansion Ratio; WU: Water Uptake; ASV: Alkali Spreading Value; AC: Amylose Content.

Further, correlation analysis between grain quality and agronomic traits of the short grain aromatic rice landraces showed that grain yield was significantly (r = 0.269**) correlated to ear bearing tiller number but not related to any of the grain quality traits (Table 2). A highest positive correlation was found between kernel length (KL) and kernel length after cooking (KLAC) (r = 0.855**) followed by volume expansion ratio and kernel length after cooking (r = 0.558**).

**Table 2:** Co-relation between grain quality and agronomic traits.

	PH	DUR	EBT	PL	YL	KL	KB	VER	KLAC	WU	ASV	AC
**PH**	-											
**DUR**	0.124*	-										
**EBT**	-0.163**	-0.079	-									
**PL**	0.503**	-0.040	-0.096	-								
**YL**	0.076	0.148*	0.269**	0.083	-							
**KL**	-0.316**	-0.050	-0.079	-0.247**	0.081	-						
**KB**	-0.022	0.034	-0.232**	-0.009	-0.015	0.090	-					
**VER**	-0.174**	0.021	0.021	-0.063	0.051	0.418**	0.100	-				
**KLAC**	-0.292**	-0.047	-0.031	-0.225**	0.042	0.855**	0.08	0.558**	-			
**WU**	0.105	0.045	0.028	-0.066	-0.069	0.072	0.050	0.032	0.047	-		
**ASV**	0.005	0.131*	0.013	-0.055	0.077	0.027	0.057	-0.031	0.133*	0.048	-	
**AC**	-0.002	0.076	-0.161*	-0.027	0.06	0.094	0.091	0.05	0.039	-0.012	0.008	-

** and * = significant at *p < 0*.*01* and *p < 0*.*05* level, respectively.

PH: Plant Height; DYR: Duration; EBT: Ear Bearing Tiller; PL: Panicl Length; YL: Yield; KL: Kernel Length; KB: Kernel Breadth; VER: Volume Expansion Ratio; KLAC: Kernel Length After Cooking; WU: Water Uptake; ASV: Alkali Spreading Value; AC: Amylose Content

**Fig 2:**
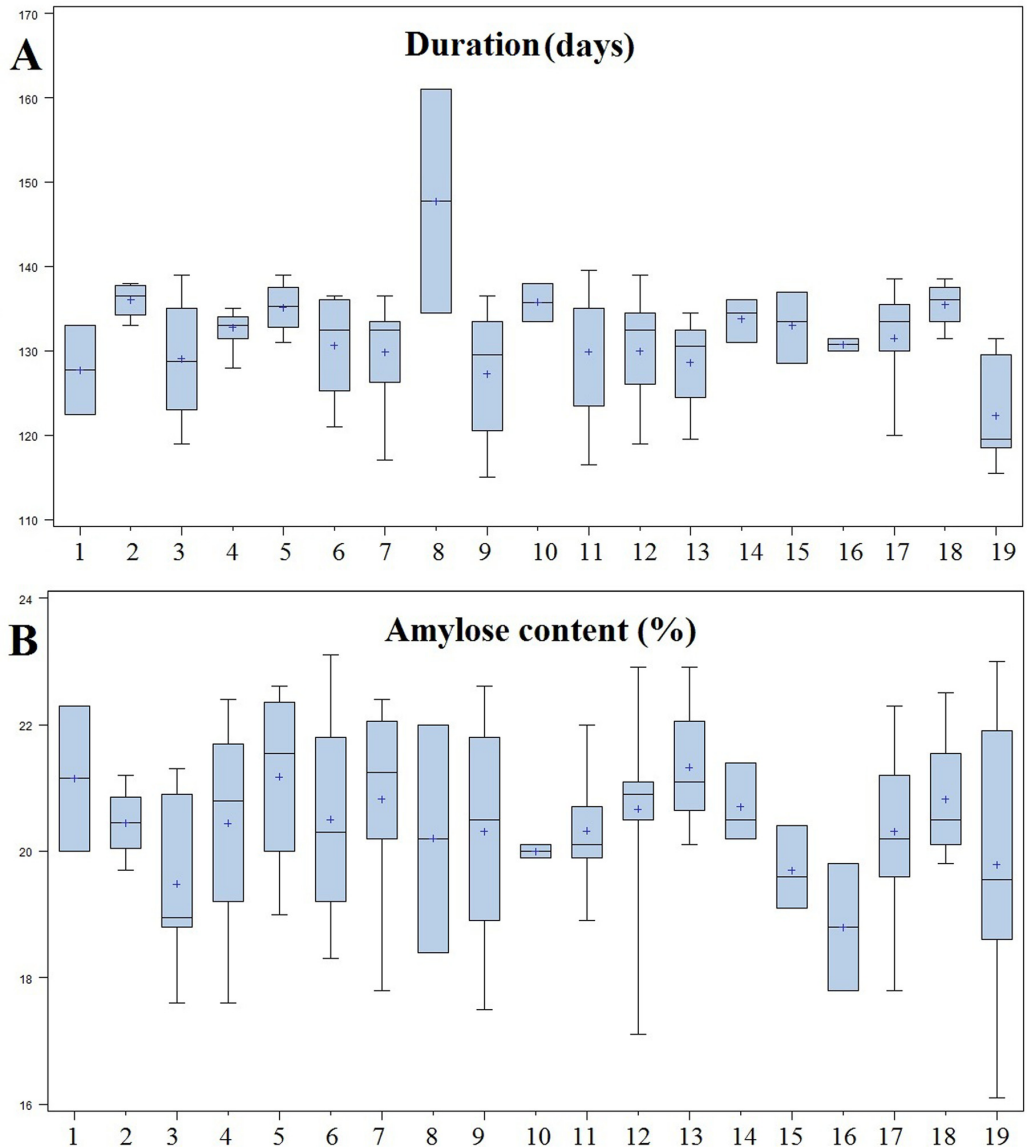
Distribution of duration and amylose content in short grain aromatic rice genotypes collected from 19 districts. Note: Numbers in the x-axis represent 19 different districts [Anugul (1), Balasore (2), Bolangir (3), Cuttack (4), Deogarh (5), Dhenkanal (6), Ganjam (7), Jajpur (8), Kalahandi (9), Kendrapara (10), Kenojhar (11), Koraput (12), Malkangiri (13), Mayurbhanj (14), Nayagarh (15), Kandhamal (16), Puri (17), Sambalpur (18) and Sundargarh (19)].

Wide variation for agronomic and grain quality traits was observed for the short grain aromatic rice genotypes in different districts (S1 Fig). Genotypes having medium tall plant height were observed in the districts of Anugul, Deogarh and Koraput. But the genotypes collected from Cuttack and Nayagarh were comparatively taller. All the districts of Odisha have grown landraces of medium to late duration except in Jajpur district where landraces were of very late duration (Fig 2A). The landraces of Nayagarh, Kandhamal and Sambalpur were high yielders (>4.0 t/ha) while genotypes from Anugul, Bolangir, Kalahandi, Keonjhar, Mayurbhanj were low yielders. Similarly, for grain quality traits, landraces with short bold grain types were prominent in most of the districts except in Anugul and Dhenkanal. For alkali spreading value, it was intermediate in range with the exception of Dhenkanal where it was high. A high variation for volume expansion ratio was detected in the short grain aromatic landraces of Mayurbhanj district. Majority of the landraces showed intermediate amylose content but landraces with low values were detected from Jajpur, Kalahandi and Kandhamal districts (Fig 2B).

### Microsatellite marker diversity

A total of 110 alleles were recorded with the 24 polymorphic SSR markers in the short grain aromatic rice landraces examined. The number of alleles ranged from 2 (RM2381, RM6839 and RM4862) to 12 (RM2705) with a mean of 4.583 alleles per locus. Further, RM8060 and RM2935 could amplify a total of 8 alleles each. The number of effective alleles (*Ne*) varied from 1.036 to 8.2993 and more than 54% of the SSR loci were with >2.0 value. With an average of 0.553, the *PIC* value varied between 0.059 (lowest) and 0.965 (highest) for the markers RM6839 and RM4838, respectively (Table 3). Markers like RM8213, RM207, RM2935, RM8020, RM206, RM2705, RM4862 and RM4838 showed high *PIC* value of more than 0.70. The polymorphic markers could reveal handful of genetic diversity among the genotypes (average = 0.494) with the Nei’s genetic diversity value (*He*) in the range of 0.034 (RM2381) to 0.880 (RM2705). Shannon’s diversity index was in line with Nei’s genetic diversity value for the tested marker set.

**Table 3:** Genetic diversity parameters calculated on SSR data.

Locus	*Na*	*Ne*	*PIC*	*Ho*	*He*	*I*
RM10864	4	2.627	0.473	0.378	0.619	1.108
RM1360	5	3.769	0.567	0.262	0.735	1.430
RM6378	5	2.413	0.597	0.412	0.586	1.088
RM207	6	3.556	0.730	0.278	0.719	1.428
RM422	3	1.155	0.124	0.865	0.134	0.295
RM186	3	1.538	0.367	0.649	0.350	0.650
RM8213	5	3.493	0.717	0.283	0.714	1.419
RM3866	6	2.355	0.587	0.422	0.575	1.140
RM4838	3	1.374	0.965	0.723	0.272	0.536
RM480	3	2.593	0.378	0.383	0.614	1.025
RM8060	8	2.966	0.657	0.335	0.663	1.492
RM2615	3	2.652	0.605	0.379	0.611	1.030
RM2381	2	1.036	0.530	0.966	0.034	0.088
RM336	5	1.452	0.310	0.605	0.302	0.639
RM80	6	3.426	0.225	0.289	0.708	1.467
RM8020	6	4.049	0.825	0.243	0.753	1.567
RM2705	12	8.299	0.851	0.117	0.880	2.241
RM6839	2	1.066	0.059	0.938	0.062	0.141
RM590	3	1.629	0.444	0.612	0.386	0.703
RM1375	3	1.197	0.190	0.835	0.164	0.357
RM206	4	1.446	0.836	0.690	0.309	0.629
RM4862	2	1.584	0.926	0.627	0.369	0.556
RM2935	8	5.35	0.819	0.184	0.813	1.847
RM3472	3	1.954	0.493	0.510	0.488	0.817
**Mean**	4.583	2.624	0.553	0.499	0.494	0.987
**St. Dev**	2.358	1.650	0.256	0.246	0.248	0.553

*Na*: number of alleles; *Ne*: number of effective alleles; *PIC*: polymorphism information content; *Ho*: expected homozygosity; *He*: Nei’s genetic diversity; *I*: Shannon’s information index.

#### Genetic relationship and population structure

**Fig 3:**
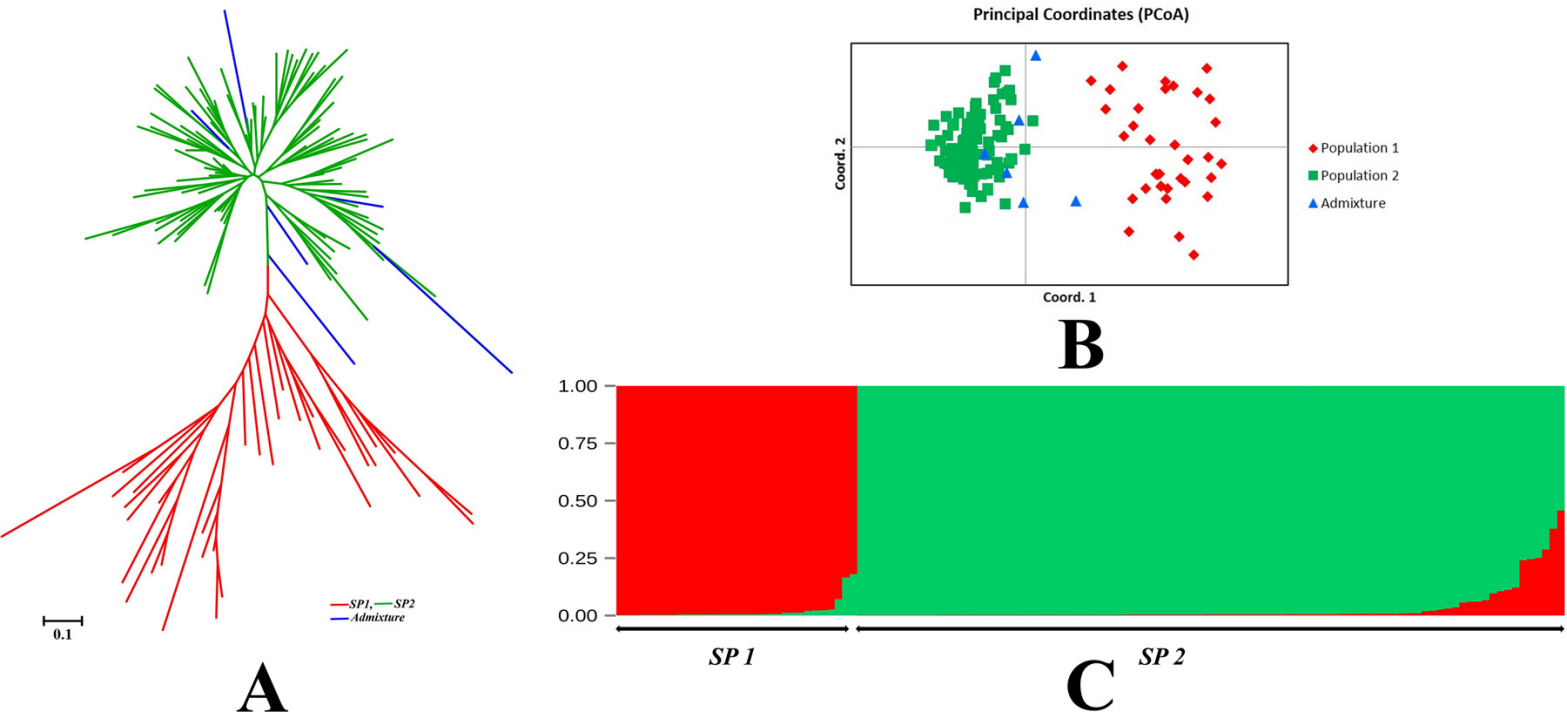
Genetic relationships and population structure of short grain aromatic rices. A: NJ dendrogram; B: Principal Co-ordinate analysis; C: Population Structure.

The pairwise Nei’s genetic distance for the short grain aromatic rice landraces ranged from 0.062 for the pair Kendumanjee—Thakurbhog to 2.821 for the pair Suman–Atmasital1 and Deulabhog3—Panasmanjee with an average of 0.811. The NJ (Neighbour-Joining) dendrogram constructed based on the pair wise genetic distance matrix grouped short grain aromatic rice landraces into two major clusters (Fig 3A). A total of 34 genotypes were grouped in cluster I whereas, 102 were grouped in cluster II. Genotypes of cluster II were closely related as compared to that of cluster I. The principal co-ordinate analysis (PCoA) revealed that 21.99% and 6.66% of the total variation was explained by the first two co-ordinates (Fig 3B). However, the first two principal co-ordinates could explain 28.65% of the variation cumulatively. Further, the population structure analysis suggested the presence of moderate population structure in the genotype set, where the highest log likelihood value was K = 2. This indicated that the entire population could be divided into two sub-populations i.e. SP 1 with a membership percentage of 27.1%, while SP 2 with 72.9% (Fig 3C). The fixation index (*F*_ST_) values of subpopulations were 0.294 and 0.187 for SP1 and SP2, respectively with an average of 0.241 (allele frequency divergence = 0.179). However, an average genetic distance of 0.397 and 0.536 were recorded between the genotypes of sub-population SP1 and SP2, respectively (Table 4). Further, having a membership percentage of <80% for any given sub- population, a total of 6 admixtures were detected. All the detected admixtures detected were from SP2 group. The NJ dendrogram, PCoA and population structure results were in line with no exceptions.

**Table 4:** Population statistics of the estimated clusters.

Population	Membership (%)	*F*_ST_	Average distances	AF divergence
SP 1	27.1	0.294	0.397	0.179
SP 2	72.9	0.187	0.536	

SP 1 and SP 2 are estimated subpopulations at K = 2; AF: allele frequency.

#### Molecular genetic diversity based on geographical districts

The genotypes were grouped according to their location of the collection and estimates were made on the genetic diversity present within a particular district. The total number of alleles and number of effective alleles were positively related to the number of genotypes collected from each district. The total number of different allele per district ranged from 1.417 (Jajpur) to 3.583 (Koraput). Similarly, number of private alleles (NPA), i.e. specific to a given landrace, of 0.042 was detected in few of the districts i.e. Anugul, Balasore, Cuttack and Ganjam followed by 0.083 in Puri. On an average, number of locally common alleles (NLA) found in < 25% of the population in the range of 0.042 in Keonjhar to 0.542 in Koraput district (Table 5). However, in districts like Jajpur, Mayurbhanj and Nayagarh, no locally common alleles were detected. The Nei’s genetic diversity value (*He*) among the genotypes representing a particular district ranged from 0.229 (Jajpur) to 0.480 (Puri). Further, the districts like Koraput and Malkangiri also detected to be having a high genetic diversity value of 0.476 and 0.460, respectively. The Shannon’s information index (*I*) corresponded well with that of Nei’s genetic diversity. The analysis of molecular variance (AMOVA) suggested that on an average, 92% variation exists within districts and 8% variation was observed among the districts (Table 6). The pairwise Nei’s unbiased genetic distance among districts ranged from 0.046 (Cuttack and Koraput) to 1.099 (Jajpur and Kandhamal) with an average of 0.295 (S4 Table).

**Table 5:** SSR based results on genetic diversity parameters—District wise.

Districts	Co-ordinates	*Na*	*Ne*	*NPA*	*NLA*	*He*	*I*
Anugul	20°47′50″N 85°1′26″E	1.542	1.467	0.042	0.125	0.255	0.379
Balasore	21.49°N 86.93°E	1.958	1.736	0.042	0.167	0.363	0.567
Bolangir	20.72°N 83.48°E	3.083	2.173	0.000	0.375	0.439	0.799
Cuttack	20.27°N 85.52°E	3.458	2.269	0.042	0.500	0.446	0.837
Deogarh	21.53°N 84.73°E	2.375	2.164	0.000	0.125	0.438	0.725
Dhenkanal	20.67°N 85.6°E	2.167	1.932	0.000	0.083	0.391	0.634
Ganjam	19.38°N 85.07°E	3.375	2.304	0.042	0.417	0.440	0.824
Jajpur	20.85°N 86.333°E	1.417	1.386	0.000	0.000	0.229	0.327
Kalahandi	20.083°N 83.2°E	2.083	1.844	0.000	0.167	0.354	0.569
Kendrapara	20.525°N 86.475°E	1.583	1.567	0.000	0.083	0.307	0.428
Keonjhar	21.63°N 85.58°E	2.167	1.747	0.000	0.042	0.309	0.521
Koraput	18.8083°N 82.7083°E	3.583	2.416	0.000	0.542	0.460	0.875
Malkangiri	18.35°N 81.90°E	3.125	2.470	0.000	0.375	0.476	0.876
Mayurbhanj	21.933°N 86.733°E	1.708	1.618	0.000	0.000	0.241	0.393
Nayagarh	20.116°N 85.01°E	1.917	1.795	0.000	0.000	0.347	0.536
Phulbani (Kandhamal)	20.47°N 84.23°E	1.542	1.511	0.000	0.125	0.271	0.384
Puri	20.47°N 84.23°E	3.458	2.525	0.083	0.375	0.480	0.901
Sambalpur	19.48°N 85.49°E	2.042	1.847	0.000	0.083	0.355	0.565
Sundargarh	21.47°N 83.97°E	2.417	1.924	0.000	0.125	0.359	0.621

*Na*: number of alleles; *Ne*: number of effective alleles; *NPA*: number of provate alleles; *NLA*: number of least common alleles; *He*: Nei’s genetic diversity; *I*: Shannon’s information index.

**Table 6:** Analysis of molecular variance of short grain aromatic rices based on SSR marker data (district wise).

Source	df	SS	MS	*F*_ST_	EV	%
Among Population	18	232.133	12.218	0.078	0.059	8
Within Population	233	1387.010	5.978	-	5.978	92
Total	251	1619.143	-	-	6.487	100

df: degree of freedom; SS: Sum of squares; MS: Mean squares; EV: Estimated variance (p>0.001).

**Fig 4:**
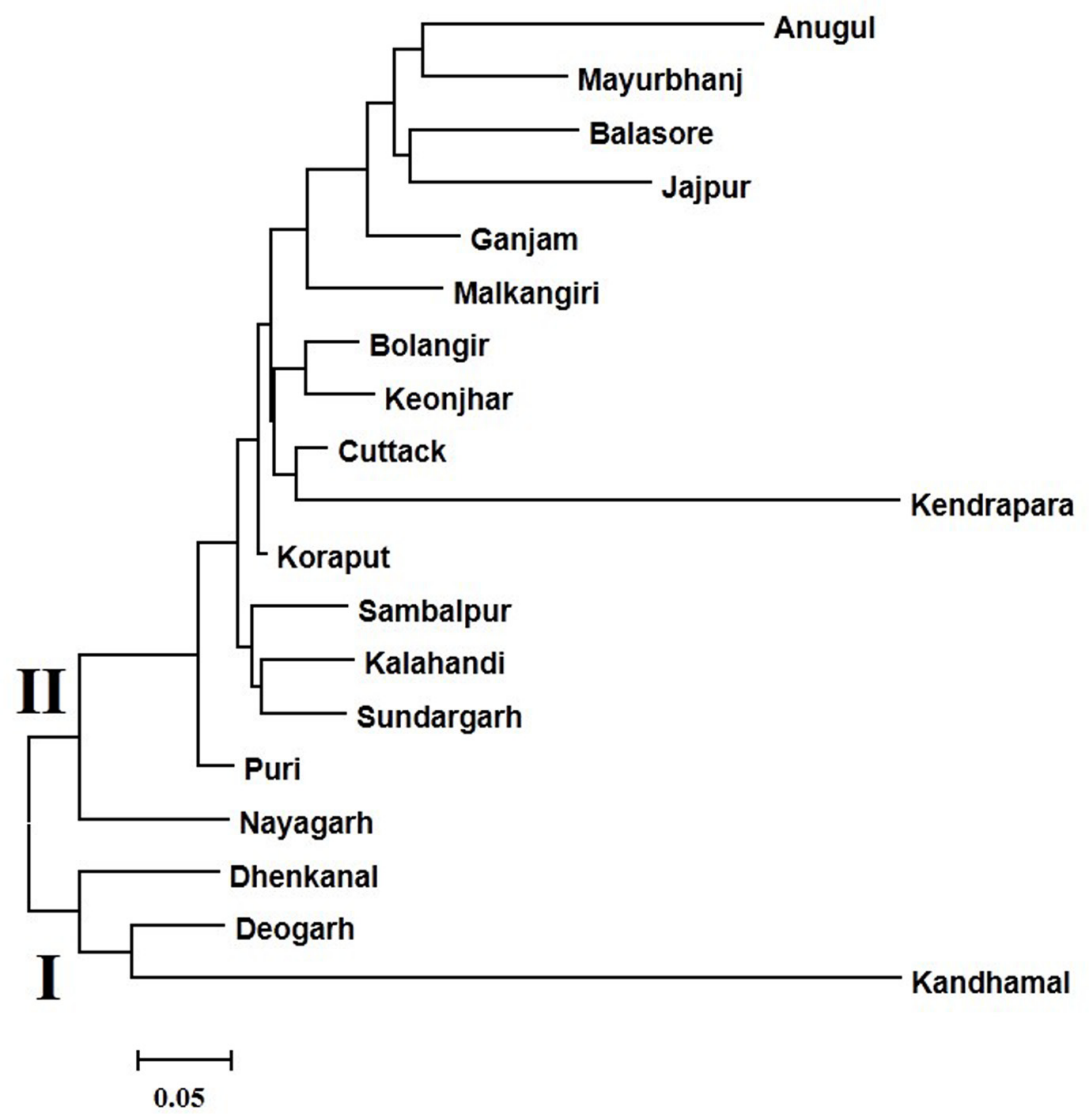
Unrooted neighbour-joining tree showing genetic relationship of short aromatic grain rices (based on SSR marker data) among 19 geographical districts.

The neighbour-joining dendrogram broadly grouped the 19 districts in two clusters i.e. I and II (Fig 4). Maximum of the districts were grouped in cluster II which contained Anugul, Mayurbhanj, Balasore, Jajpur, Ganjam, Malkangiri, Bolangir, Keonjhar, Cuttack, Kendrapara, Koraput, Sambalpur, Kalahandi, Sundargarh Puri and Nayagarh. Cluster I contained only three districts i.e. Deogarh, Dhenkanal and Kandhamal.

#### Phylogenetic relationships at chloroplast level

The PCR assays performed to amplify the cpDNA (chloroplast DNA) Pst-12 fragment ORF100 revealed the presence of the 69bp deletion in 14 (12 genotypes from Odisha, one Kalanamak accession from Uttar Pradesh and Gopalbhog from Madhya Pradesh) genotypes indicating their classification as indica types while the rest of the genotypes were of *japonica* type having the product without deletion. The target PS1D region was amplified with PD1D, a specific primer and the amplified product was sequenced directly after purification. The pairwise alignment showed that all the sequences were highly identical to each other with an average identity of 95% (range 90–95%) at the DNA level. Out of 552 total numbers of sites, 31 segregating sites were detected in the landraces. Average pairwise nucleotide diversity (*Π*) and silent Watterson’s nucleotide diversity estimator (θ_w_) were 0.919 and 0.895, respectively. The nucleotide diversity between the PS1D regions of different accessions was compared and the results showed that the wild progenitors and landraces were highly variable. Tests of selection and neutrality (Tajima’s D) were performed to assess the evolutionary processes influencing each gene region. The values of Tajima’s D (-0.674) were negative and significant (Table 7).

**Table 7:** Nucleotide polymorphism parameters of *PS1D* region.

Region	Sample size	Segregating sites	Nucleotide diversity *(Π)*	Watterson nucleotide diversity (θ_w_)	Number of haplotype (h)	Haplotype diversity	Tajima’s d
*PD1D*	140	31	0.919	0.895	33	0.440	-0.0674

**Fig 5:**
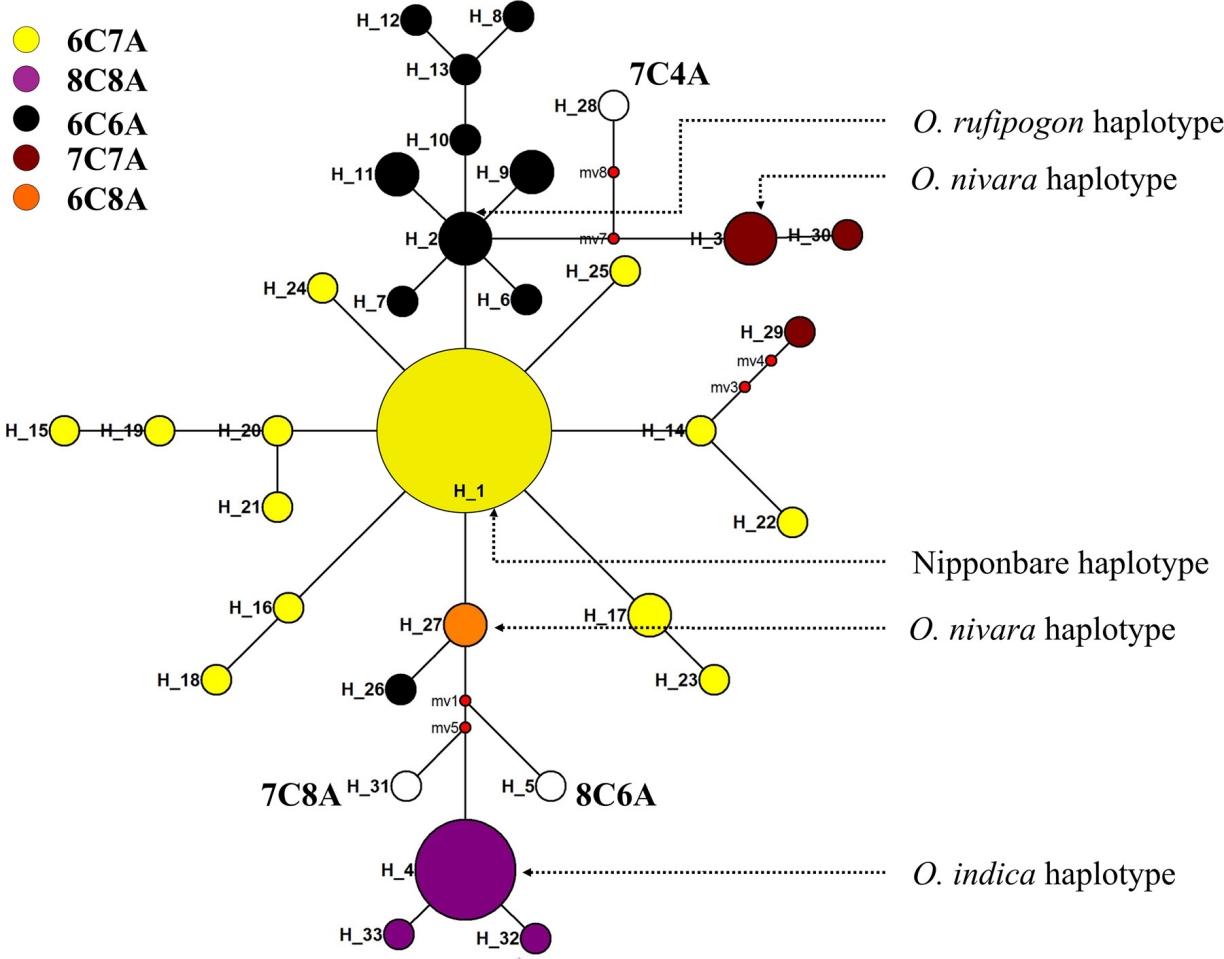
Haplotype tree depicting different haplotypes among the short grain aromatic rices based on nucleotide polymorphism of the PS1D region.

Based on C/A repeats, 8 different plastid sub types were detected in the short grain aromatic rice landraces with *O*. *japonica* (Nipponbare) subtype (6C7A) was detected in 100 genotypes followed by *O*. *rufipogon* (KF428978) subtype (6C6A) in 13 genotypes and *O*. *indica* (93–11) sub type (8C8A) in 14 genotypes, including one of the Kalanamak accession from Uttar Pradesh and Gopalbhog from Madhya Pradesh. Two of the *O*. *nivara* subtypes were observed in the genotype set i.e. 7C7A subtype in 2 genotypes (Chatianaki and Atmasital) and 6C8A subtype in another two genotypes (Mahulakuchi and Mahulkuchi). Further, three of the genotypes contained different subtypes for C/A repeat i.e. Jayaphul, Karpurkali and Deulabhog-3 with 8C6A, 7C4A and 7C8A types, respectively. All the genotypes were grouped into 33 different haplotypes with a haplotype diversity of 0.440. The major haplotype Hap1 contained 88 genotypes along with the reference genotype Nipponbare followed by Hap4 which contained 13 genotypes including *O*. *indica* types. Two of the genotypes i.e. Leelabati and Kalagiri were in one group viz. Hap2 along with *O*. *rufipogon*. *O*. *nivara* formed a separate haplotype group (Hap3) containing two more (Chatianaki and Atmasital-1) genotypes (S5 Table). Further, a haplotype tree was constructed based on sequence data for diagrammatic representation of differentiation of genotypes is given in Fig 5. The haplotype tree represented the number of haplotypes and the SNP position which separated one haplotype from the other. Haplotype frequency could be inferred by size of the circles, for example the larger the haplotype circle contained maximum number of genotypes.

## Discussion

Assessment of genetic variation in specialty rices of a particular region/state has implications for conservation of these neglected heritage material and can help in unlocking genes controlling valuable traits for their utilization in rice improvement [22]. The state of Odisha, is one of the major rice producing states in India housing a large assembly of indigenous rice genotypes [23], particularly of short grain aromatic rices which have not been classified systematically.

### Characterization of landraces for agro-morphological and grain quality traits

The aromatic short grain rices characterized in the present study showed a high range of variability for various agronomic and quality traits, like plant height, duration, yield, kernel length and breadth, water uptake, alkali spreading value and amylose content. Use of morphological traits for preliminary evaluation and assessing genetic diversity in aromatic rice cultivars was reported by Hien *et al*. [24]. Similarly, Mathure *et al*. [25] reported high diversity in aromatic rice landraces from Maharashtra. The extent of genetic variability in these aromatic rices was quite high as compared to the earlier report in upland rice collection from the same state [26].

Generally aromatic rices are tall, take longer to mature, prone to lodging and low yielders [3]. Similar observations were recorded in the present set of short grain aromatic rices with all the genotypes had a duration of >145 days. However, nine landraces having medium tall plant height were identified in this study, which can be further improved by mobilization of yield and yield related traits into their genetic background using different breeding approaches. Number of reproductive tillers and panicle length has direct effect on yield per plant [27,28]. In the present study, 26 landraces having longer panicles (>30cm) and 10 genotypes having high number of reproductive tillers (> 10) were identified, along with 18 genotypes with a yield potential of > 4t/ha which could serve as useful starting material for aromatic rice improvement. Multilocation evaluation of these lines in different locations over years could probably result in identification and release of superior genotypes [29]. Almost all the genotypes examined in this study possess short bold (SB) and/or medium bold (MB) grain in conformity with the earlier reports [3, 25] Rice quality, a complex trait, determined by several factors, is an important aspect of determining market value and consumers’ preference for aromatic rices [30,31]. For instance, amylose content is related to the texture and palatability of the cooked rice [32,33]. Aromatic rices are preferred in domestic and international markets for their superior grain and cooking quality features [34]. In the present study, though high level of variation for grain quality traits was seen, majority of the genotypes were in the intermediate range for different quality traits. Yield enhancement of these high quality aromatic rices can help in higher market gain for the poor farmers of eastern India [35]. The statistical relationships between various Agronomic traits observed in this study is similar to earlier observations [25, 36, 37–42].

### Molecular characterization

#### Genetic diversity at nuclear level

In our study, a total of 110 alleles were detected with an average of 4.583 per locus (24 SSR markers), which is significantly higher than earlier reports [5, 43, 44, 45]. In contrast, existence of lower level of genetic diversity is also known [46, 47]. The high PIC value detected in our study could be due to the use of SSR markers that amplify the hyper variable region of rice chromosomes. Similar, high allelic diversity (*PIC* = 0.652) in the aromatic rice collection of West Bengal, a neighboring state of Odisha has also been reported [48] suggesting that eastern part of India houses diverse aromatic rice germplasm.

Rice landraces, being selected over years of natural and artificial impacts, possesses rich genetic diversity and broad plasticity to adapt to various agro-ecological environments [49]. Different domestication events are the primary factors for heterogeneous nature of landraces that arises due to insertion, deletion and introgression at genetic level [50]. This situation present in the landraces widens the possibility of rice landraces to survive in different natural extremes. However, genetic diversity is directly related to the available heterozygosity in a population. Since, rice in India has undergone severe bottlenecks, identification of a core set of germplasm that represents the total diversity and utilizing it for rice improvement is a necessity. In the collection of population studied, the average Nei’s genetic diversity (*He* = 0.494) was higher than the reports on other rice collections from Assam [51] and north-east India [52] and from Taiwan [53] in Taiwan.

#### Population structure and genetic relationships

The pairwise distance measures reflect the level of genetic relationship and relatedness among the genotypes. In this study, a lowest pairwise genetic distance was detected between the entries, Kendumanjee–Thakurbhog, indicating genetic difference between them. However, few of the genotypes having similar local names collected from same/different area were genetically different that could be due to accumulation of different genetic constituent(s) over the period of selection in different area [54]. In addition, physical seed exchanges and inadvertent naming errors, and farmers’ selection practices to suit their local cultivation practices could explain very high genetic variation observed among the genotypes studied is possible. Similar levels of high genetic variability among the rice genotypes of North-Korea [55] and Brazil [56], respectively. With the introduction of high yielding and hybrid varieties, though yield has been enhanced, the genetic base of rice cultivars has narrowed down [57,58]. In the present collection of aromatic rices, a moderate population structure was detected as found in North-Eastern [49], a diverse origin [59] and in the North Korean [55] collections. The aromatic rice from Odisha was differentiated into two sub-populations (SP1 and SP2). Majority of the genotypes clustered in SP2 group (72.9%) suggesting their enormous importance and further studies collectively with aromatic rice landraces from nearby states can throw light on their possible origin and migration patterns. Further, most (8) of the aromatic short grain rices collected from other states were grouped in SP2, the major cluster indicating close relationship between aromatic short grain rices of Odisha and other parts of the country.

#### Diversity of landraces at different geographic locations

Farmers exert their own selection to choose the genotypes of their liking and to suit for their cultivation practices and traditionally have maintained them since ages. Hence, the set of rice landraces available in one area genetically differs from those in other area [3, 60]. Therefore, estimation of genetic diversity of individual districts considering them as a sub-population along with those from the neighbouring districts in a region would provide more meaningful insights into the overall crop diversity available in a given area.

Domestication events, farmers’ preference and natural selection has a direct relationship with the level of adaptation of landraces to biotic and abiotic stresses and as well as for agronomic traits [61]. In the present study, a particular range of morphological and grain quality traits were confined to a certain geographic area. For example, prevalence of medium tall plant stature in Anugul, Deogarh and Koraput districts, tall plant stature in Cuttack and Nayagarh, very late duration genotypes in Jajpur, high alkali spreading in the genotypes of Dhenkanal, low amylose content in the genotypes of Jajpur, Kalahandi and Kandhamal suggest the types of preferences in different regions (districts) of Odisha. Similar preferences for rice landraces of Thailand [5] and Korea [55] have been noticed.

Western districts of Odisha which includes Eastern-Ghats are mainly occupied by tribal communities and are recognized as biodiversity hot-spots [62]. Aromatic rice landraces collected from these particular areas showed high genetic diversity than those from the districts of eastern parts as was detected by us in case of qualitative morphological traits of the same set of genotypes in an earlier study [63]. The poor adaptability of improved varieties in the difficult terrain and the continued inclination of the tribal to cultivate native landraces in these regions may reason out the preservation of natural variability in western Odisha till date. Further, a low level (8%) of the total variation observed among districts could be due to frequent seed exchange between the farmers of Odisha [5].

### Differentiation of landraces at chloroplast DNA level

The inconsistency displayed by the molecular markers in drawing phylogenetic relationships might be due to genetic recombination of nuclear molecular markers [64]. Achieving consistent results with high reproducibility, while establishing phylogenetic relationships, is shown to be possible by using chloroplast markers [64, 65]. The polymorphism pattern exhibited by the hyper variable PS1D region of rice chloroplast [11] could provide insights into maternal origin of aromatic short grain rice landraces of Odisha. In the present study, the sequence variations of the genotypes based on C/A repeat of PS1D region could detect 8 different plastid subtypes i.e. 6C7A, 6C6A, 8C8A, 7C7A, 6C8A, 8C6A, 7C4A and 7C8A. The variation is higher in number than reported by Prathepha [66] who could detect only 6 plastid subtypes in Thailand’s upland and low land rice accessions. The C/A repeats of rice PS1D sequence have been studied previously in both wild and landraces [11,55,64,66,67]. Takahashi *et al*. [64] has discriminated different wild rice groups based on their C/A repeats. Establishment of phylogenetic relationship of cultivated rice/ landraces with the wild ancestors based on plastid subtype is quite relevant in the context that the plastid region of chloroplast is highly conserved in the course of evolution [11]. Characterisation of aromatic rices grown in Japan using chloroplast and mitochondrial primers differentiated them into tropical japonica and temperate japonica [68], as these organelle markers provide more information over a larger evolutionary history of crops [69]. In the present study, though the collection of landraces are from one particular geographical region i.e. Odisha, 33 different haplotypes were identified. Most of the genotypes collected from Odisha (100 out of 126) and other states (8 out of 10) were of *japonica* sub-type. This result hold true with the concept of close evolutionary relationship between aromatic group and *japonica* [70]. The identification of 14 genotypes with *indica* subtype justifies the intermediate status of aromatic rices between *indica* and *japonica* [70,71]. The two different Kalanamak accessions collected from Uttar Pradesh showed difference for their plastid sub-type (one *japonica* and one *indica*). Since Kalanamak is highly preferred for its grain quality and aroma, characterization of different Kalanamak accessions could be relevant to understand their evolutionary pattern. The haplotype groups correspond well with the C/A repeats of the genotypes. However, some of the genotypes having same C/A repeats of japonica/indica formed separate clusters which could be due to the possibility of higher mutational rate in landraces over the period of evolution and domestication [67]. The interesting observation of novel repeats in Jayaphul (8C6A), Karpurkali (7C4A) and Deulabhog-3 (7C8A) warrants further investigation on their complete chloroplast genome so as to understand the evolution of such repeat types. Evolution and domestication of rice has been always a complex phenomenon to study. However, *O*. *rufipogon* is believed to be the wild progenitor of domesticated rice [72] and *O*. *nivara* shares close association with *O*. *rufipogon* [73,74]. In an earlier study on the variability in the ORF 100 region of the wild progenitors and the cultivated forms of Eastern India [75], though novel variants were not observed in either of the populations, the frequency of *japonica* allele was very high in the landraces. The haplotype tree constructed in the present study suggests the possibility of origin of *nivara* and *japonica* type alleles from *O*. *rufipogon* and *indica* type alleles from *japonica*. Since, origin of *indica* types is not clear, it can assumed that origin of the chloroplast genome is independent in different short grain aromatic rices.

## Conclusion

Indigenous aromatic short grain rices hold enormous importance for the local farmers and consumers. Though the population studied and the geographic area were small, the variability was high and extensive hybridization followed by selection during domestication must have played a role in generation of novel variability in these aromatic rices, known to be the intermediate forms between *japonica* and *indica*. Extensive studies at organelle (chloroplast and mitochondria) and ribosome level of these aromatic short grain aromatic rice landraces could provide further insights into their origin.

## Supporting Information

S1 Fig Distribution of agronomic and grain quality traits in short grain aromatic rice genotypes collected from 19 districts. (TIF)

S1 Table List of short grain aromatic rice landraces used in the present study and region of their collection. (DOCX)

S2 Table List of primers used in this study. (DOCX)

S3 Table Detailed agro-morphological and grain quality parameters of short grain aromatic rices. (DOCX)

S4 Table Pair-wise Nei’s unbiased genetic distance of short grain aromatic rices based on SSR markers of 19 geographical districts. (DOCX)

S5 Table The frequency distribution of different haplotypes. (DOCX)
