# Supplementary figures and images for: Nuclear and Chloroplast DNA Variation Provides Insights into Population Structure and Multiple Origin of Native Aromatic Rices of Odisha, India
Source: PLoS One. 2016 Sep 6;11(9):e0162268. doi: 10.1371/journal.pone.0162268 (PMC5012674; doi:10.1371/journal.pone.0162268)

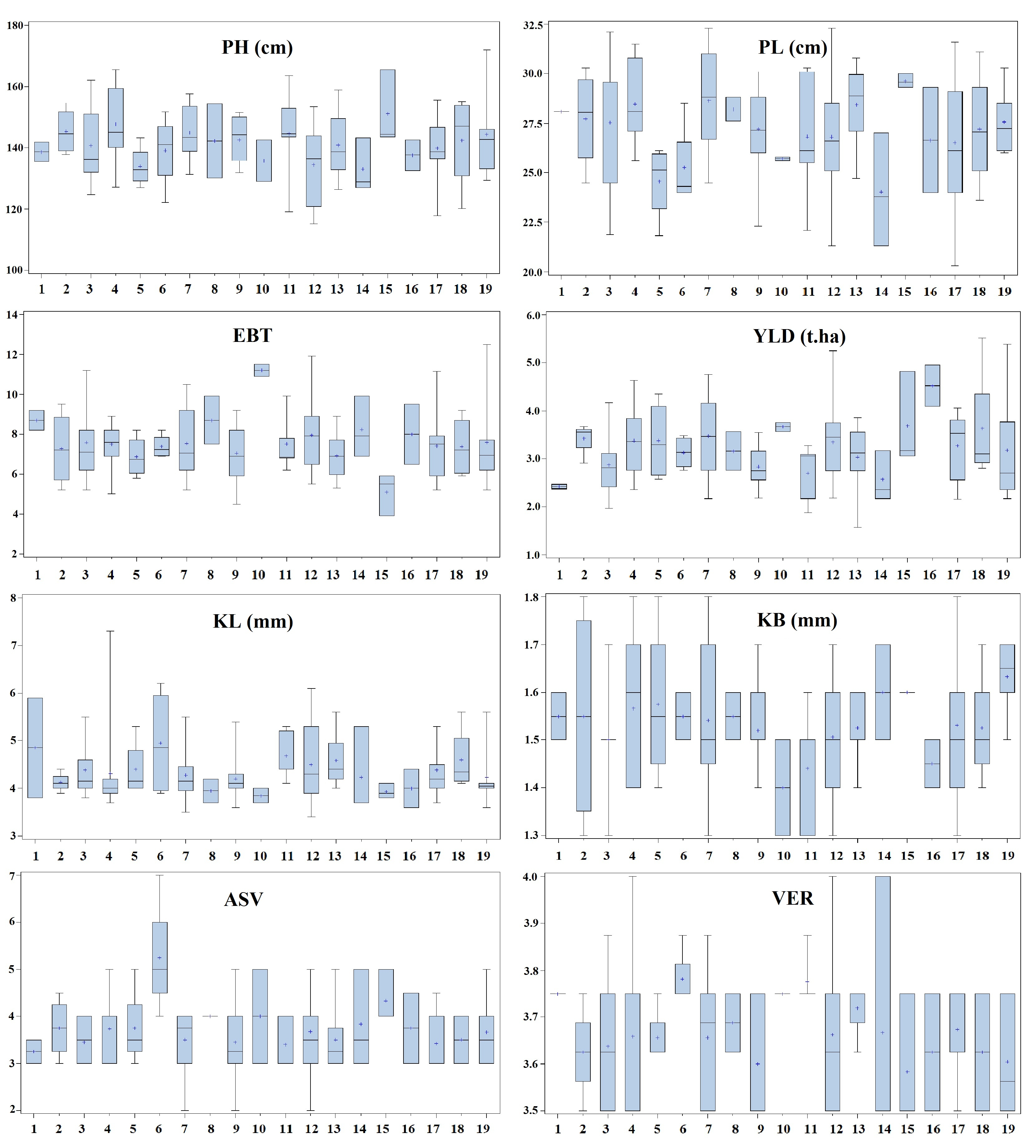

Supplement: S1 Fig — (TIF) [file pone.0162268.s001.tif]
