# Supplementary material for: Nuclear and Chloroplast DNA Variation Provides Insights into Population Structure and Multiple Origin of Native Aromatic Rices of Odisha, India
Source: PLoS One. 2016 Sep 6;11(9):e0162268. doi: 10.1371/journal.pone.0162268 (PMC5012674; doi:10.1371/journal.pone.0162268)
Supplement: S1 Table — (DOCX) [file pone.0162268.s002.docx]

S1 Table. Short grain aromatic rice landraces used in the present study and region of their collection

| **Sl. No.** | **Accession number** | **Varieties** | **Area of collection** | **State** |
| --- | --- | --- | --- | --- |
| 1 | 44119 | Baluchi | Dhenkanal | Orissa |
| 2 | 44120 | Acharmati-1 | Bolangir | Orissa |
| 3 | 44121 | Acharmati-2 | Bolangir | Orissa |
| 4 | 44122 | Basaya bhog | Sundargarh | Orissa |
| 5 | 44123 | Basanasapuri | Puri | Orissa |
| 6 | 44124 | Basua bhog-1 | Anugul | Orissa |
| 7 | 44125 | Baukunja | Cuttack | Orissa |
| 8 | 44126 | Basasa phool | Bolangir | Orissa |
| 9 | 44127 | Badsabhog | Bolangir | Orissa |
| 10 | 44128 | Bhatagundi | Koraput | Orissa |
| 11 | 44129 | Bhadraka Basumati | Balasore | Orissa |
| 12 | 44130 | Basumati-1 | Cuttack | Orissa |
| 13 | 44131 | Baiganamanji | Bhawanipatna | Orissa |
| 14 | 44132 | Basaparijata | Kalahandi | Orissa |
| 15 | 44133 | Basanapuri | Puri | Orissa |
| 16 | 44134 | Basubhog | Koraput | Orissa |
| 17 | 44135 | Basanadhan | Koraput | Orissa |
| 18 | 44136 | Basanaphula | Cuttack | Orissa |
| 19 | 44137 | Chatianaki | Cuttack | Orissa |
| 20 | 44138 | Deulabhog-1 | Puri | Orissa |
| 21 | 44139 | Deulabhog-2 | Puri | Orissa |
| 22 | 44140 | Dhusara | Cuttack | Orissa |
| 23 | 44141 | Dubrajsena | Koraput | Orissa |
| 24 | 44142 | Durgabhog | Keonjhar | Orissa |
| 25 | 44143 | Dhurabahila | Koraput | Orissa |
| 26 | 44144 | Deulabhog-3 | Koraput | Orissa |
| 27 | 44145 | Dangar Basumati | Koraput | Orissa |
| 28 | 44146 | Dubraj | Koraput | Orissa |
| 29 | 44147 | Ganjamlocal-1 | Ganjam | Orissa |
| 30 | 44148 | Ganjamlocal-2 | Ganjam | Orissa |
| 31 | 44149 | Ganjeikali | Dhenkanal | Orissa |
| 32 | 44150 | Jaiphool | Bolangir | Orissa |
| 33 | 44151 | Jhillipanjar | Cuttack | Orissa |
| 34 | 44152 | Jala | Keonjhar | Orissa |
| 35 | 44153 | Jhingisali | Balasore | Orissa |
| 36 | 44154 | Kalajeera-1 | Mayurbhanj | Orissa |
| 37 | 44155 | Karpurkali | Ganjam | Orissa |
| 38 | 44156 | Kalikati-1 | Kalahandi | Orissa |
| 39 | 44157 | Kala krishna | Kalahandi | Orissa |
| 40 | 44158 | Kukudajata | Koraput | Orissa |
| 41 | 44159 | Koiamba-543 | Koraput | Orissa |
| 42 | 44160 | Kanakchampa | Keonjhar | Orissa |
| 43 | 44161 | Karpurabasa | Koraput | Orissa |
| 44 | 44162 | Krishnabhog | Puri | Orissa |
| 45 | 44163 | Kalajiri-1 | Puri | Orissa |
| 46 | 44164 | Karpurazeera | Kalahandi | Orissa |
| 47 | 44165 | Kendumanjee | Koraput | Orissa |
| 48 | 44166 | Laxmibilas-1 | Deogarh | Orissa |
| 49 | 44167 | Laxmibilas-2 | Sambalpur | Orissa |
| 50 | 44168 | Leelabati | Balasore | Orissa |
| 51 | 44169 | Lektimachi-1 | Malkangiri | Orissa |
| 52 | 44170 | Lektimasi | Malkangiri | Orissa |
| 53 | 44171 | Lektimachi-2 | Malkangiri | Orissa |
| 54 | 44172 | Laser | Malkangiri | Orissa |
| 55 | 44173 | Mahulakuchi | Malkangiri | Orissa |
| 56 | 44174 | Magura selectioin | Ganjam | Orissa |
| 57 | 44175 | Manas | Puri | Orissa |
| 58 | 44176 | Manasi | Puri | Orissa |
| 59 | 44177 | Mahulkuchi | Malkangiri | Orissa |
| 60 | 44178 | Nalidhan | Cuttack | Orissa |
| 61 | 44179 | Nanu | Anugul | Orissa |
| 62 | 44180 | Pirima | Koraput | Orissa |
| 63 | 44181 | Panasmanjee | Malkangiri | Orissa |
| 64 | 44182 | Sunsuniasunaphul | Deogarh | Orissa |
| 65 | 44183 | Badaguda | Deogarh | Orissa |
| 66 | 44184 | Benugopal | Sambalpur | Orissa |
| 67 | 44185 | Jayaphul | Sundargarh | Orissa |
| 68 | 44186 | Benubhog | Mayurbhanj | Orissa |
| 69 | 44187 | Samleibhog-1 | Sundargarh | Orissa |
| 70 | 44188 | Bhuinsasal | Deogarh | Orissa |
| 71 | 44189 | Kalajira | Dhenkanal | Orissa |
| 72 | 44190 | Laxmikajol | Keonjhar | Orissa |
| 73 | 44191 | Shantibhog | Puri | Orissa |
| 74 | 44192 | Sujata | Puri | Orissa |
| 75 | 44193 | Thakursuna | Cuttack | Orissa |
| 76 | 44194 | Suman | Cuttack | Orissa |
| 77 | 44195 | Thakur bhog | Puri | Orissa |
| 78 | 44196 | Atmasital-1 | Koraput | Orissa |
| 79 | 44197 | Nagri | Koraput | Orissa |
| 80 | 44198 | Pipalbasa | Sambalpur | Orissa |
| 81 | 44199 | Samleibhog-2 | Sundargarh | Orissa |
| 82 | 44200 | Kalazeera | Dhenkanal | Orissa |
| 83 | 44201 | Laxmibilas-3 | Sambalpur | Orissa |
| 84 | 44202 | Basnadhan-1 | Sundargarh | Orissa |
| 85 | 44203 | Kalaziri | Ganjam | Orissa |
| 86 | 44204 | Basumati-2 | Sundargarh | Orissa |
| 87 | 44205 | Parijatak | Ganjam | Orissa |
| 88 | 44206 | Magura | Ganjam | Orissa |
| 89 | 44207 | Gadakakudinga | Phulbani | Orissa |
| 90 | 44208 | Gangabali | Ganjam | Orissa |
| 91 | 44209 | Karpurakranti | Ganjam | Orissa |
| 92 | 44210 | Phulabani local | Phulbani | Orissa |
| 93 | 44211 | Kalajeera-2 | Ganjam | Orissa |
| 94 | 44212 | Kalagiri | Cuttack | Orissa |
| 95 | 44213 | Nadiarasa | Cuttack | Orissa |
| 96 | 44214 | Kendragali | Cuttack | Orissa |
| 97 | 44215 | Saragadhuli | Cuttack | Orissa |
| 98 | 44216 | Karpurakanta | Cuttack | Orissa |
| 99 | 44217 | Basumati-3 | Kendrapara | Orissa |
| 100 | 44218 | Basuabhog-2 | Kendrapara | Orissa |
| 101 | 44219 | Garmatia | Puri | Orissa |
| 102 | 44220 | Krisna bhog | Puri | Orissa |
| 103 | 44221 | Tulasi basa | Nayagarh | Orissa |
| 104 | 44222 | Kalatulasi | Nayagarh | Orissa |
| 105 | 44223 | Kalajeera-3 | Nayagarh | Orissa |
| 106 | 44224 | Batakarua | Keonjhar | Orissa |
| 107 | 44225 | Basumati-4 | Jajpur | Orissa |
| 108 | 44226 | Kalajiri-2 | Ganjam | Orissa |
| 109 | 44227 | Suetpotato | Jajpur | Orissa |
| 110 | 44228 | Maharaji | Kalahandi | Orissa |
| 111 | 44229 | Laktimachi | Koraput | Orissa |
| 112 | 44230 | Karpurakali | Ganjam | Orissa |
| 113 | 44231 | Pimpudibasa | Mayurbhanj | Orissa |
| 114 | 44232 | Atmasital-2 | Malkangiri | Orissa |
| 115 | 44233 | Kalajeera-4 | Koraput | Orissa |
| 116 | 44234 | Nadiaphool | Cuttack | Orissa |
| 117 | 44235 | Jawaphool | Bolangir | Orissa |
| 118 | 44236 | Kalikati-2 | Bhawanipatna | Orissa |
| 119 | 44237 | Basnadhan-2 | Bhawanipatna | Orissa |
| 120 | 44238 | Morllu | Bhawanipatna | Orissa |
| 121 | 44239 | Basanaparijata | Bhawanipatna | Orissa |
| 122 | 44240 | Lilabati | Balasore | Orissa |
| 123 | 44241 | Ramabana Basmati | Bolangir | Orissa |
| 124 | 44242 | Kalkati | Bolangir | Orissa |
| 125 | 44243 | Nadiakata | Bolangir | Orissa |
| 126 | 44244 | Kalakanhu | Bolangir | Orissa |
