# Supplementary material for: Nuclear and Chloroplast DNA Variation Provides Insights into Population Structure and Multiple Origin of Native Aromatic Rices of Odisha, India
Source: PLoS One. 2016 Sep 6;11(9):e0162268. doi: 10.1371/journal.pone.0162268 (PMC5012674; doi:10.1371/journal.pone.0162268)
Supplement: S2 Table — (DOCX) [file pone.0162268.s003.docx]

S2 Table. Primers used for analysis of genetic variability in short grain aromatic rice collections

| **Marker** | **Ch.** | **Repeat motif** | **Forward Primer** | **Reverse primer** | **Exp.product size (bp)** | **Annealing temp.** |
| --- | --- | --- | --- | --- | --- | --- |
| **Nuclear SSRs** | | | | | | |
| RM1360 | 1 | (AG)25 | ttacctcaggctcttcaggc | agaagtgagcaatcatggcc | 154 | 55 |
| RM10864 | 1 | (GT)27 | gaggtgagtgagacttgacagtgc | gctcatcatccaaccacagtcc | 239 | 57 |
| RM207 | 2 | (CT)25 | ccattcgtgagaagatctga | cacctcatcctcgtaacgcc | 118 | 55 |
| RM6378 | 2 | (GAA)19 | atagggtgggtgtgctgaac | tgcacaaaactgcaggtctc | 167 | 55 |
| RM422 | 3 | (AG)30 | ttcaacctgcatccgctc | ccatccaaatcagcaacagc | 385 | 55 |
| RM186 | 3 | (CGG)5 | tcctccatctcctccgctcccg | gggcgtggtggccttcttcgtc | 124 | 61 |
| RM3866 | 4 | (GA)29 | agttggtcatctaccagagc | gatcttcttgcctcagaaag | 161 | 55 |
| RM8213 | 4 | (TC)10 | agcccagtgatacaaagatg | gcgaggagataccaagaaag | 177 | 55 |
| RM480 | 5 | (AC)30 | gctcaagcattctgcagttg | gcgcttctgcttattggaag | 225 | 55 |
| RM 4838 | 5 | (TA)28 | cattcagatgaagcaacacagacc | ccaaatacaatcgatggagtgacc | 124 | 55 |
| RM8060 | 6 | (AT)27 | ggttgtgctgaatactgtccataagc | caggtaaccggtgaagatgtcg | 147 | 55 |
| RM2615 | 6 | (AT)30 | cagagtgctttagacaatca | aaattggtaagagattctgc | 164 | 55 |
| RM2381 | 7 | (AT)26 | aacctcaaatatttaaactc | gctagagaaaatagagaaac | 142 | 55 |
| RM336 | 7 | (CTT)18 | cttacagagaaacggcatcg | gctggtttgtttcaggttcg | 154 | 55 |
| RM80 | 8 | (TCT)25 | ttgaaggcgctgaaggag | catcaacctcgtcttcaccg | 142 | 55 |
| RM8020 | 8 | (TA)20(GA)19 | atcctcgatgaattgtatat | gaagaggtgtacatgaataa | 167 | 55 |
| RM2705 | 9 | (AT)33 | ataaagtatcaaatcaactg | atttaattgaaggaatataa | 155 | 55 |
| RM6839 | 9 | (TCT)17 | ctactgttgcaggcttgcag | cagaggaggagatcgagagg | 104 | 50 |
| RM590 | 10 | (TCT)10 | catctccgctctccatgc | ggagttggggtcttgttcg | 137 | 55 |
| RM1375 | 10 | (AG)31 | ctacacgcgcaaactctgtc | atgaaggtctaggctgcacc | 180 | 50 |
| RM206 | 11 | (CT)21 | cccatgcgtttaactattct | cgttccatcgatccgtatgg | 147 | 55 |
| RM4862 | 11 | (TA)28 | caactttctggcataaacta | tggtgaaagatatttcagac | 164 | 55 |
| RM2935 | 12 | (AT)39 | cagcaaatttgttacttatg | tgctatgtttttttataacg | 165 | 55 |
| RM3472 | 12 | (CT)21 | atcgcaagaactccgtgaag | cgcttttgagctcgcctc | 215 | 55 |
| **Chloroplast specific marker** | | | | | | |
| PD1D | - | - | aaagatctagatttcgtaaacaacatagaggaagaa | atctgcagcatttaaaagggttctgaggttgaatcat | 550 | 55 |

Ch. Chromosome
