# Supplementary material for: Nuclear and Chloroplast DNA Variation Provides Insights into Population Structure and Multiple Origin of Native Aromatic Rices of Odisha, India
Source: PLoS One. 2016 Sep 6;11(9):e0162268. doi: 10.1371/journal.pone.0162268 (PMC5012674; doi:10.1371/journal.pone.0162268)
Supplement: S3 Table — (DOCX) [file pone.0162268.s004.docx]

S3 Table. Detailed agro-morphological and grain quality parameters of short grain aromatic rices

| **Genotype** | **PH (cm)** | **DFF (days)** | **PL (cm)** | **EBT** | **YLD (t/ha)** | **KL (mm)** | **KB (mm)** | **KLAC (mm)** | **VER** | **WU** | **ASV** | **AC (%)** |
| --- | --- | --- | --- | --- | --- | --- | --- | --- | --- | --- | --- | --- |
| Baluchi | 142.25 | 137 | 24.60 | 6.95 | 3.48 | 5.68 | 1.46 | 10.52 | 3.75 | 113 | 5 | 20.45 |
| Acharmati-1 | 134.55 | 127 | 27.80 | 7.90 | 2.41 | 4.38 | 1.54 | 8.12 | 3.50 | 60 | 3 | 18.86 |
| Acharmati-2 | 155.00 | 135 | 32.10 | 8.20 | 2.57 | 4.02 | 1.50 | 8.08 | 3.50 | 110 | 4 | 17.58 |
| Basaya bhog | 142.15 | 119 | 30.30 | 5.20 | 3.04 | 3.99 | 1.73 | 8.21 | 3.63 | 115 | 4 | 21.86 |
| Basanasapuri | 146.60 | 132 | 26.50 | 7.50 | 4.05 | 5.01 | 1.58 | 8.79 | 3.63 | 88 | 3 | 21.91 |
| Basua bhog-1 | 135.45 | 133 | 28.10 | 8.20 | 2.47 | 3.83 | 1.47 | 7.37 | 3.75 | 110 | 3 | 22.29 |
| Baukunja | 127.15 | 133 | 27.60 | 8.50 | 3.36 | 7.26 | 1.52 | 12.30 | 4.00 | 115 | 4 | 21.65 |
| Basasa phool | 150.95 | 123 | 28.10 | 6.20 | 2.85 | 4.22 | 1.39 | 7.68 | 3.50 | 108 | 3 | 18.80 |
| Badsabhog | 149.65 | 135 | 28.40 | 6.20 | 3.11 | 3.77 | 1.55 | 7.73 | 3.50 | 130 | 4 | 19.00 |
| Bhatagundi | 120.75 | 128 | 21.30 | 7.90 | 2.37 | 4.09 | 1.32 | 8.85 | 3.50 | 103 | 4 | 21.85 |
| Bhadraka Basumati | 154.50 | 138 | 29.10 | 6.20 | 2.90 | 3.95 | 1.40 | 8.65 | 3.50 | 98 | 4 | 20.40 |
| Basumati-1 | 145.05 | 131 | 30.80 | 5.00 | 2.72 | 3.90 | 1.68 | 8.95 | 3.75 | 90 | 3 | 19.96 |
| Baiganamanji | 151.45 | 133 | 26.80 | 6.90 | 2.56 | 3.56 | 1.53 | 6.95 | 3.50 | 120 | 3 | 20.66 |
| Basaparijata | 146.75 | 137 | 28.80 | 8.90 | 2.77 | 5.39 | 1.53 | 10.41 | 3.75 | 113 | 4 | 17.50 |
| Basanapuri | 137.15 | 134 | 24.00 | 11.15 | 3.37 | 4.49 | 1.43 | 8.70 | 3.75 | 100 | 5 | 19.60 |
| Basubhog | 136.40 | 122 | 28.80 | 11.90 | 3.17 | 3.81 | 1.67 | 8.55 | 3.75 | 108 | 4 | 21.10 |
| Basanadhan | 146.45 | 126 | 32.30 | 7.20 | 2.96 | 3.97 | 1.42 | 7.93 | 3.63 | 85 | 4 | 21.54 |
| Basanaphula | 140.10 | 132 | 26.00 | 8.20 | 3.85 | 5.22 | 1.56 | 9.25 | 3.75 | 103 | 3 | 20.84 |
| Chatianaki | 140.65 | 134 | 27.10 | 7.50 | 2.37 | 3.92 | 1.42 | 8.10 | 3.50 | 120 | 4 | 18.04 |
| Deulabhog-1 | 120.30 | 120 | 23.60 | 5.90 | 2.16 | 4.34 | 1.53 | 8.37 | 3.50 | 115 | 3 | 21.01 |
| Deulabhog-2 | 137.85 | 136 | 23.60 | 7.90 | 3.81 | 3.96 | 1.70 | 8.20 | 3.75 | 130 | 4 | 19.20 |
| Dhusara | 159.25 | 132 | 26.80 | 8.90 | 3.36 | 4.40 | 1.42 | 9.00 | 3.75 | 120 | 3 | 19.19 |
| Dubrajsena | 140.85 | 135 | 27.50 | 9.20 | 3.56 | 3.41 | 1.44 | 6.89 | 3.50 | 118 | 5 | 20.45 |
| Durgabhog | 119.05 | 124 | 22.10 | 9.90 | 1.88 | 5.21 | 1.48 | 10.15 | 3.88 | 120 | 3 | 18.85 |
| Dhurabahila | 143.85 | 134 | 26.30 | 8.90 | 2.17 | 5.26 | 1.45 | 10.35 | 3.63 | 110 | 4 | 17.14 |
| Deulabhog-3 | 140.25 | 139 | 24.50 | 6.05 | 3.74 | 5.75 | 1.69 | 10.65 | 3.75 | 115 | 5 | 22.91 |
| Dangar Basumati | 115.15 | 136 | 24.60 | 6.90 | 3.45 | 5.72 | 1.55 | 11.08 | 3.75 | 110 | 4 | 20.88 |
| Dubraj | 119.85 | 124 | 26.10 | 6.00 | 4.29 | 5.50 | 1.60 | 10.35 | 3.75 | 100 | 4 | 20.54 |
| Ganjamlocal-1 | 151.45 | 131 | 29.60 | 10.20 | 3.01 | 3.50 | 1.31 | 8.00 | 3.75 | 103 | 3 | 21.76 |
| Ganjamlocal-2 | 157.60 | 134 | 31.10 | 7.20 | 3.28 | 4.15 | 1.75 | 7.85 | 3.50 | 130 | 4 | 22.24 |
| Ganjeikali | 139.90 | 130 | 24.00 | 6.90 | 2.91 | 4.01 | 1.51 | 8.89 | 3.75 | 105 | 5 | 23.14 |
| Jaiphool | 125.05 | 128 | 21.85 | 11.20 | 4.16 | 5.52 | 1.33 | 9.28 | 3.50 | 105 | 4 | 21.31 |
| Jhillipanjar | 139.85 | 132 | 29.60 | 7.85 | 3.55 | 4.04 | 1.38 | 8.26 | 3.75 | 100 | 3 | 20.81 |
| Jala | 143.40 | 140 | 25.50 | 6.80 | 3.27 | 5.28 | 1.30 | 8.85 | 3.75 | 100 | 4 | 20.13 |
| Jhingisali | 148.85 | 136 | 27.00 | 9.50 | 3.56 | 4.14 | 1.32 | 8.26 | 3.63 | 110 | 5 | 21.16 |
| Kalajeera-1 | 126.90 | 135 | 21.30 | 6.90 | 2.37 | 5.28 | 1.73 | 10.32 | 4.00 | 120 | 4 | 20.18 |
| Karpurkali | 133.80 | 135 | 24.50 | 7.20 | 3.75 | 5.52 | 1.65 | 9.89 | 3.75 | 123 | 4 | 21.96 |
| Kalikati-1 | 150.10 | 121 | 26.50 | 6.90 | 3.55 | 4.44 | 1.55 | 8.46 | 3.50 | 118 | 2 | 18.85 |
| Kala krishna | 131.85 | 135 | 29.20 | 5.90 | 2.75 | 4.08 | 1.58 | 7.62 | 3.50 | 148 | 4 | 22.63 |
| Kukudajata | 153.30 | 128 | 28.00 | 8.75 | 2.34 | 3.54 | 1.55 | 7.90 | 3.50 | 110 | 3 | 21.33 |
| Koiamba-543 | 128.45 | 134 | 25.10 | 5.75 | 2.36 | 4.30 | 1.60 | 9.20 | 3.63 | 83 | 5 | 21.05 |
| Kanakchampa | 144.50 | 135 | 26.10 | 7.80 | 2.17 | 4.37 | 1.60 | 9.04 | 3.75 | 148 | 3 | 20.68 |
| Karpurabasa | 119.30 | 136 | 25.60 | 8.90 | 3.16 | 4.42 | 1.47 | 8.78 | 3.75 | 128 | 3 | 20.71 |
| Krishnabhog | 152.95 | 139 | 29.10 | 7.70 | 3.75 | 4.03 | 1.56 | 7.90 | 3.63 | 100 | 4 | 17.84 |
| Kalajiri-1 | 144.75 | 134 | 28.45 | 8.90 | 2.77 | 3.74 | 1.38 | 8.06 | 3.50 | 113 | 3 | 21.15 |
| Karpurazeera | 150.85 | 134 | 22.30 | 8.20 | 3.16 | 3.99 | 1.48 | 7.52 | 3.50 | 148 | 3 | 19.60 |
| Kendumanjee | 140.55 | 119 | 29.60 | 9.20 | 3.76 | 3.87 | 1.55 | 8.03 | 3.75 | 120 | 4 | 20.23 |
| Laxmibilas-1 | 126.90 | 135 | 21.80 | 6.30 | 3.84 | 3.96 | 1.42 | 7.95 | 3.63 | 110 | 3 | 21.01 |
| Laxmibilas-2 | 154.95 | 139 | 31.10 | 5.90 | 3.05 | 4.48 | 1.45 | 8.72 | 3.75 | 120 | 3 | 19.83 |
| Leelabati | 140.10 | 133 | 30.30 | 5.20 | 3.67 | 4.43 | 1.79 | 9.20 | 3.75 | 108 | 3 | 20.46 |
| Lektimachi-1 | 136.00 | 130 | 24.70 | 5.30 | 2.75 | 4.38 | 1.57 | 8.65 | 3.75 | 103 | 3 | 22.19 |
| Lektimasi | 141.35 | 123 | 29.00 | 6.90 | 3.28 | 5.57 | 1.55 | 11.20 | 3.75 | 130 | 4 | 20.89 |
| Lektimachi-2 | 158.75 | 133 | 30.30 | 6.90 | 3.85 | 5.22 | 1.50 | 9.65 | 3.75 | 120 | 3 | 20.43 |
| Laser | 135.25 | 126 | 28.80 | 7.20 | 3.35 | 4.24 | 1.41 | 8.75 | 3.75 | 80 | 4 | 20.05 |
| Mahulakuchi | 126.35 | 135 | 25.60 | 8.20 | 3.75 | 4.43 | 1.53 | 9.27 | 3.75 | 105 | 5 | 22.94 |
| Magura selectioin | 142.70 | 127 | 27.00 | 9.20 | 2.77 | 3.89 | 1.44 | 8.55 | 3.63 | 115 | 4 | 20.08 |
| Manas | 117.75 | 121 | 20.30 | 6.85 | 2.56 | 5.33 | 1.76 | 9.90 | 3.75 | 118 | 3 | 18.95 |
| Manasi | 135.15 | 128 | 30.30 | 5.20 | 2.26 | 5.17 | 1.40 | 8.95 | 3.63 | 120 | 3 | 21.41 |
| Mahulkuchi | 153.20 | 132 | 29.60 | 6.20 | 2.76 | 4.18 | 1.63 | 8.22 | 3.75 | 95 | 3 | 21.91 |
| Nalidhan | 156.70 | 135 | 30.80 | 8.20 | 4.64 | 4.16 | 1.63 | 7.50 | 3.50 | 115 | 3 | 21.00 |
| Nanu | 141.80 | 123 | 28.10 | 9.20 | 2.37 | 5.90 | 1.60 | 10.60 | 3.75 | 98 | 4 | 20.01 |
| Pirima | 136.25 | 129 | 28.10 | 9.70 | 5.25 | 4.25 | 1.44 | 7.99 | 3.50 | 98 | 4 | 21.03 |
| Panasmanjee | 130.40 | 120 | 28.60 | 5.75 | 1.57 | 4.71 | 1.46 | 8.29 | 3.63 | 130 | 3 | 21.11 |
| Sunsuniasunaphul | 131.55 | 139 | 26.10 | 7.20 | 4.34 | 4.29 | 1.54 | 8.21 | 3.63 | 90 | 5 | 19.01 |
| Badaguda | 134.00 | 136 | 25.80 | 8.20 | 2.57 | 4.03 | 1.64 | 8.07 | 3.75 | 93 | 4 | 22.56 |
| Benugopal | 120.25 | 137 | 23.60 | 9.20 | 5.52 | 5.64 | 1.73 | 10.50 | 3.75 | 93 | 3 | 20.41 |
| Jayaphul | 171.95 | 130 | 27.65 | 7.70 | 5.38 | 5.58 | 1.59 | 9.43 | 3.75 | 140 | 4 | 22.96 |
| Benubhog | 128.95 | 136 | 23.80 | 7.90 | 2.17 | 3.74 | 1.50 | 7.36 | 3.50 | 108 | 5 | 20.46 |
| Samaleibhog-1 | 133.15 | 120 | 26.00 | 6.20 | 2.17 | 4.06 | 1.58 | 8.04 | 3.75 | 115 | 3 | 18.55 |
| Bhuinsasal | 143.10 | 131 | 24.50 | 5.80 | 2.75 | 5.27 | 1.80 | 9.23 | 3.63 | 95 | 4 | 22.08 |
| Kalajira | 151.65 | 136 | 28.50 | 8.20 | 3.36 | 3.90 | 1.57 | 8.90 | 3.88 | 115 | 4 | 18.25 |
| Laxmikajol | 163.35 | 135 | 30.30 | 6.85 | 3.10 | 4.06 | 1.51 | 8.20 | 3.75 | 113 | 4 | 22.00 |
| Shantibhog | 136.45 | 138 | 26.10 | 6.90 | 3.75 | 4.24 | 1.47 | 7.86 | 3.63 | 108 | 3 | 22.26 |
| Sujata | 138.65 | 131 | 24.00 | 10.20 | 3.96 | 4.21 | 1.30 | 9.29 | 3.75 | 120 | 4 | 19.88 |
| Thakursuna | 146.05 | 135 | 28.10 | 6.20 | 3.83 | 3.97 | 1.78 | 7.93 | 3.75 | 125 | 4 | 20.59 |
| Suman | 159.15 | 128 | 29.00 | 7.20 | 3.75 | 4.21 | 1.58 | 8.39 | 3.50 | 105 | 5 | 21.71 |
| Thakur bhog | 155.30 | 134 | 31.60 | 5.20 | 3.53 | 4.49 | 1.64 | 8.41 | 3.75 | 125 | 3 | 19.81 |
| Atmasital-1 | 117.45 | 135 | 24.10 | 7.90 | 3.75 | 4.58 | 1.52 | 8.83 | 3.63 | 123 | 2 | 19.50 |
| Nagri | 146.65 | 133 | 28.50 | 8.90 | 3.56 | 3.62 | 1.45 | 7.10 | 3.50 | 120 | 3 | 19.24 |
| Pipalbasa | 152.70 | 132 | 27.50 | 8.20 | 3.16 | 4.17 | 1.50 | 7.73 | 3.50 | 125 | 4 | 22.49 |
| Samleibhog-2 | 143.15 | 116 | 26.80 | 6.90 | 2.37 | 4.01 | 1.67 | 7.49 | 3.50 | 95 | 4 | 19.66 |
| Kalazeera | 122.20 | 121 | 24.00 | 7.50 | 2.76 | 6.16 | 1.59 | 10.90 | 3.75 | 158 | 7 | 20.05 |
| Laxmibilas-3 | 141.35 | 136 | 26.60 | 6.20 | 2.80 | 4.13 | 1.45 | 7.50 | 3.50 | 85 | 4 | 20.56 |
| Basnadhan-1 | 146.05 | 132 | 28.50 | 7.00 | 2.37 | 3.56 | 1.67 | 7.34 | 3.50 | 120 | 5 | 16.08 |
| Kalaziri | 146.20 | 137 | 26.60 | 5.20 | 4.10 | 4.27 | 1.74 | 7.73 | 3.50 | 98 | 4 | 21.53 |
| Basumati-2 | 129.35 | 119 | 26.10 | 12.50 | 3.77 | 4.08 | 1.49 | 7.25 | 3.50 | 110 | 3 | 19.44 |
| Parijatak | 143.05 | 122 | 31.00 | 9.20 | 4.20 | 3.85 | 1.50 | 8.45 | 3.50 | 138 | 4 | 21.00 |
| Magura | 131.35 | 133 | 28.00 | 5.90 | 2.75 | 4.64 | 1.67 | 8.76 | 3.63 | 103 | 3 | 20.63 |
| Gadakakudinga | 142.60 | 132 | 29.30 | 6.50 | 4.09 | 4.38 | 1.39 | 8.35 | 3.75 | 110 | 3 | 17.83 |
| Gangabali | 143.45 | 133 | 25.60 | 6.90 | 2.17 | 4.18 | 1.55 | 8.52 | 3.75 | 100 | 2 | 20.30 |
| Karpurakranti | 136.70 | 117 | 30.10 | 10.50 | 4.75 | 4.29 | 1.33 | 9.61 | 3.88 | 95 | 4 | 17.75 |
| Phulabani local | 132.55 | 130 | 24.00 | 9.50 | 4.95 | 3.64 | 1.49 | 7.86 | 3.50 | 110 | 5 | 19.81 |
| Kalajeera-2 | 140.85 | 126 | 32.30 | 6.90 | 2.47 | 4.87 | 1.75 | 9.33 | 3.75 | 140 | 3 | 22.11 |
| Kalagiri | 140.80 | 134 | 31.50 | 7.60 | 4.15 | 3.93 | 1.68 | 7.57 | 3.50 | 100 | 4 | 22.40 |
| Nadiarasa | 136.55 | 133 | 27.60 | 8.20 | 2.57 | 4.24 | 1.53 | 8.66 | 3.63 | 118 | 5 | 17.63 |
| Kendragali | 165.40 | 134 | 30.80 | 6.90 | 2.96 | 4.06 | 1.44 | 7.60 | 3.50 | 135 | 4 | 21.43 |
| Saragadhuli | 160.45 | 133 | 28.10 | 6.50 | 2.95 | 3.69 | 1.66 | 8.91 | 3.75 | 138 | 4 | 21.86 |
| Karpurakanta | 156.95 | 134 | 27.50 | 8.90 | 3.76 | 3.77 | 1.69 | 7.93 | 3.75 | 130 | 4 | 19.06 |
| Basumati-3 | 142.55 | 138 | 25.60 | 10.90 | 3.77 | 3.74 | 1.55 | 9.66 | 3.75 | 115 | 5 | 19.90 |
| Basuabhog-2 | 129.05 | 134 | 25.80 | 11.50 | 3.57 | 4.04 | 1.33 | 7.60 | 3.75 | 90 | 3 | 20.11 |
| Garmatia | 155.40 | 136 | 31.10 | 5.50 | 4.05 | 3.99 | 1.53 | 7.91 | 3.75 | 85 | 4 | 20.21 |
| Krisna bhog | 140.00 | 130 | 26.10 | 7.50 | 2.57 | 4.15 | 1.58 | 7.96 | 3.75 | 120 | 4 | 20.80 |
| Tulasi basa | 144.35 | 137 | 29.60 | 5.50 | 4.82 | 3.92 | 1.56 | 7.78 | 3.75 | 110 | 4 | 20.36 |
| Kalatulasi | 143.40 | 129 | 30.00 | 3.90 | 3.17 | 4.11 | 1.63 | 8.35 | 3.50 | 65 | 5 | 19.08 |
| Kalajeera-3 | 165.45 | 134 | 29.30 | 5.90 | 3.06 | 3.81 | 1.56 | 7.59 | 3.50 | 88 | 4 | 19.61 |
| Batakarua | 152.90 | 117 | 30.10 | 6.20 | 3.06 | 4.40 | 1.30 | 8.21 | 3.75 | 113 | 3 | 19.91 |
| Basumati-4 | 154.25 | 135 | 28.80 | 9.90 | 3.56 | 4.23 | 1.47 | 8.15 | 3.75 | 135 | 4 | 18.40 |
| Kalajiri-2 | 156.40 | 134 | 31.00 | 6.50 | 4.73 | 4.03 | 1.46 | 8.47 | 3.75 | 105 | 4 | 22.43 |
| Suetpotato | 130.05 | 161 | 27.60 | 7.50 | 2.76 | 3.68 | 1.60 | 7.00 | 3.63 | 108 | 4 | 21.99 |
| Maharaji | 135.75 | 121 | 26.00 | 6.50 | 3.45 | 3.94 | 1.55 | 8.16 | 3.75 | 105 | 5 | 22.33 |
| Laktimachi | 130.10 | 135 | 26.60 | 6.50 | 4.25 | 6.15 | 1.50 | 11.30 | 4.00 | 100 | 3 | 21.05 |
| Karpurakali | 155.40 | 133 | 26.80 | 5.50 | 3.64 | 4.10 | 1.46 | 8.10 | 3.50 | 135 | 4 | 18.08 |
| Pimpudibasa | 143.15 | 131 | 27.00 | 9.90 | 3.17 | 3.71 | 1.59 | 7.30 | 3.50 | 105 | 3 | 21.36 |
| Atmasital-2 | 145.55 | 133 | 30.80 | 8.90 | 2.97 | 4.01 | 1.48 | 8.00 | 3.63 | 110 | 4 | 21.10 |
| Kalajeera-4 | 148.80 | 122 | 28.80 | 5.50 | 2.75 | 4.16 | 1.43 | 9.14 | 3.75 | 70 | 4 | 20.81 |
| Nadiaphool | 142.80 | 135 | 25.60 | 6.90 | 2.76 | 3.86 | 1.51 | 7.94 | 3.50 | 135 | 5 | 20.36 |
| Jawaphool | 136.75 | 123 | 29.50 | 7.70 | 2.76 | 4.04 | 1.53 | 8.26 | 3.75 | 143 | 4 | 17.94 |
| Kalikati-2 | 144.40 | 120 | 28.80 | 4.50 | 2.74 | 4.10 | 1.45 | 7.30 | 3.50 | 110 | 3 | 20.34 |
| Basnadhan-2 | 136.00 | 130 | 27.50 | 7.90 | 2.57 | 4.02 | 1.40 | 8.05 | 3.50 | 103 | 3 | 17.90 |
| Morllu | 143.90 | 129 | 30.10 | 5.50 | 2.55 | 4.33 | 1.66 | 8.17 | 3.75 | 105 | 4 | 21.54 |
| Basanaparijata | 134.00 | 115 | 26.00 | 9.20 | 2.18 | 4.20 | 1.48 | 8.00 | 3.75 | 100 | 4 | 21.81 |
| Lilabati | 137.70 | 138 | 24.50 | 8.20 | 3.56 | 4.14 | 1.68 | 7.40 | 3.63 | 135 | 4 | 19.73 |
| Ramabana Basmati | 124.75 | 130 | 23.30 | 5.50 | 2.16 | 5.44 | 1.67 | 9.86 | 3.88 | 125 | 3 | 20.94 |
| Kalkati | 162.10 | 139 | 30.10 | 5.20 | 1.96 | 4.12 | 1.55 | 9.90 | 3.75 | 113 | 4 | 21.05 |
| Nadiakata | 135.80 | 132 | 24.50 | 6.50 | 3.74 | 4.62 | 1.48 | 9.30 | 3.75 | 100 | 4 | 20.38 |
| Kalakanhu | 132.10 | 119 | 29.60 | 11.20 | 2.97 | 3.76 | 1.48 | 7.64 | 3.75 | 88 | 3 | 18.93 |
| Nua Kalajeera | 143.35 | 153 | 28.80 | 9.00 | 3.41 | - | - | - | - | - | - | - |
| Nua Dhusara | 140.65 | 153 | 27.15 | 8.45 | 3.22 | - | - | - | - | - | - | - |
| Nua Chinikamini | 145.50 | 142 | 28.25 | 8.50 | 3.43 | - | - | - | - | - | - | - |
| KetekiJoha | 131.65 | 146 | 32.35 | 9.15 | 3.96 | - | - | - | - | - | - | - |
| Geetanjali | 116.55 | 135 | 33.20 | 8.65 | 3.62 | - | - | - | - | - | - | - |
| **Mean** | **141.12** | **131.34** | **27.42** | **7.57** | 3.23 | **4.37** | **1.53** | **8.58** | **3.66** | **111** | **4** | **20.42** |
| **SE** | **2.06** | **2.93** | **1.39** | **0.51** | **27.14** | **0.67** | **0.12** | **1.03** | **0.13** | **17** | **1** | **1.43** |
| **LSD 5%** | **5.75** | **8.21** | **3.88** | **1.43** | **75.94** | - | - | - | - | - | - | - |
| **CV 5%** | **2.10** | **3.20** | **7.20** | **9.60** | **11.90** | - | - | - | - | - | - | - |

PH: Plant height; DFF: Days to 50% flowering; PL: Panicle length; EBT: Ear bearing tiller; YLD: Yield; KL: Kernel length; KB: Kernel breadth; KLAC: Kernel length after cooking; VER: Volume expansion ratio; WU: Water Uptake; ASV: Alkali spreading value; AC: Amylose content
