# Supplementary material for: Nuclear and Chloroplast DNA Variation Provides Insights into Population Structure and Multiple Origin of Native Aromatic Rices of Odisha, India
Source: PLoS One. 2016 Sep 6;11(9):e0162268. doi: 10.1371/journal.pone.0162268 (PMC5012674; doi:10.1371/journal.pone.0162268)
Supplement: S4 Table — (DOCX) [file pone.0162268.s005.docx]

S4 Table. Pair-wise Nei’s unbiased genetic distance of short grain aromatic rices based on SSR markers of 19 geographical districts

|  | **1** | **2** | **3** | **4** | **5** | **6** | **7** | **8** | **9** | **10** | **11** | **12** | **13** | **14** | **15** | **16** | **17** | **18** | **19** |
| --- | --- | --- | --- | --- | --- | --- | --- | --- | --- | --- | --- | --- | --- | --- | --- | --- | --- | --- | --- |
| **Anugul** | *** |  |  |  |  |  |  |  |  |  |  |  |  |  |  |  |  |  |  |
| **Balasore** | 0.293 | *** |  |  |  |  |  |  |  |  |  |  |  |  |  |  |  |  |  |
| **Bolangir** | 0.326 | 0.195 | *** |  |  |  |  |  |  |  |  |  |  |  |  |  |  |  |  |
| **Cuttack** | 0.273 | 0.175 | 0.068 | *** |  |  |  |  |  |  |  |  |  |  |  |  |  |  |  |
| **Deogarh** | 0.504 | 0.448 | 0.275 | 0.269 | *** |  |  |  |  |  |  |  |  |  |  |  |  |  |  |
| **Dhenkanal** | 0.485 | 0.404 | 0.284 | 0.283 | 0.153 | *** |  |  |  |  |  |  |  |  |  |  |  |  |  |
| **Ganjam** | 0.270 | 0.169 | 0.148 | 0.116 | 0.367 | 0.360 | *** |  |  |  |  |  |  |  |  |  |  |  |  |
| **Jajpur** | 0.353 | 0.219 | 0.251 | 0.258 | 0.542 | 0.523 | 0.221 | *** |  |  |  |  |  |  |  |  |  |  |  |
| **Kalahandi** | 0.418 | 0.246 | 0.130 | 0.128 | 0.289 | 0.261 | 0.187 | 0.294 | *** |  |  |  |  |  |  |  |  |  |  |
| **Kendrapara** | 0.519 | 0.508 | 0.370 | 0.341 | 0.422 | 0.453 | 0.458 | 0.525 | 0.429 | *** |  |  |  |  |  |  |  |  |  |
| **Keonjhar** | 0.298 | 0.202 | 0.065 | 0.088 | 0.350 | 0.327 | 0.127 | 0.196 | 0.126 | 0.403 | *** |  |  |  |  |  |  |  |  |
| **Koraput** | 0.277 | 0.169 | 0.061 | 0.046 | 0.234 | 0.259 | 0.084 | 0.226 | 0.080 | 0.339 | 0.072 | *** |  |  |  |  |  |  |  |
| **Malkangiri** | 0.304 | 0.226 | 0.149 | 0.136 | 0.321 | 0.328 | 0.159 | 0.223 | 0.143 | 0.427 | 0.181 | 0.113 | *** |  |  |  |  |  |  |
| **Mayurbhanj** | 0.260 | 0.186 | 0.184 | 0.147 | 0.439 | 0.471 | 0.128 | 0.222 | 0.256 | 0.564 | 0.143 | 0.156 | 0.236 | *** |  |  |  |  |  |
| **Nayagarh** | 0.474 | 0.385 | 0.241 | 0.260 | 0.216 | 0.201 | 0.289 | 0.317 | 0.243 | 0.543 | 0.214 | 0.190 | 0.315 | 0.360 | *** |  |  |  |  |
| **Kandhamal** | 0.884 | 1.021 | 0.527 | 0.645 | 0.463 | 0.515 | 0.775 | 1.099 | 0.536 | 0.770 | 0.750 | 0.548 | 0.572 | 0.979 | 0.590 | *** |  |  |  |
| **Puri** | 0.342 | 0.260 | 0.091 | 0.087 | 0.207 | 0.235 | 0.167 | 0.317 | 0.103 | 0.375 | 0.124 | 0.054 | 0.124 | 0.238 | 0.192 | 0.420 | *** |  |  |
| **Sambalpur** | 0.384 | 0.235 | 0.114 | 0.118 | 0.275 | 0.292 | 0.203 | 0.263 | 0.105 | 0.425 | 0.115 | 0.067 | 0.162 | 0.252 | 0.235 | 0.520 | 0.117 | *** |  |
| **Sundargarh** | 0.314 | 0.217 | 0.114 | 0.113 | 0.299 | 0.242 | 0.179 | 0.273 | 0.094 | 0.494 | 0.100 | 0.076 | 0.147 | 0.234 | 0.192 | 0.646 | 0.097 | 0.102 | *** |
