# Supplementary material for: Nuclear and Chloroplast DNA Variation Provides Insights into Population Structure and Multiple Origin of Native Aromatic Rices of Odisha, India
Source: PLoS One. 2016 Sep 6;11(9):e0162268. doi: 10.1371/journal.pone.0162268 (PMC5012674; doi:10.1371/journal.pone.0162268)
Supplement: S5 Table — (DOCX) [file pone.0162268.s006.docx]

S4 Table. The frequency distribution of different haplotypes

| **Haplotype** | **Name of genotypes with *PD1D* alleles** | | |
| --- | --- | --- | --- |
| Hap1 | Nipponbare, Bhatagundi, Durgabhog, Jhillipanjar, Kalikati, Acharmati1, Acharmati2, Basayabhog, Basanasapuri, Basuabhog1, Baukunja, Badsabhog, Baiganamanji, Basaparijata, Basanapuri, Basanaphula, Deulabhog1, Dubrajsena, Dhurabahila, DangarBasumati, Ganjamlocal1, Jaiphool, Jala, Jhingisali, Kalajeera1, Basnadhan1, Kanakchampa, Lilabati, Samaleibhog1, Kukudajata, Krishnabhog, Kendumanjee, Laxmibilas1, Lektimachi1, Lektimasi, Laser, Maguraselection, Manas, Manasi, Nalidhan, Pirima, Panasmanjee, Badaguda, Benubhog, Bhuinsasal, Kalajira, Sujata, Thakurbhog, Nagri, Pipalbasa, Samleibhog2, Laxmibilas3, Parijatak, Gadakakudinga, Gangabali, Kalajeera2, Kendragali, Saragadhuli, Basuabhog2, Garmatia, Krisnabhog, Kalatulasi, Kalajeera3, Batakarua, Basumati4, Kalajiri2, Maharaji, Laktimachi, Karpurakali, Pimpudibasa, Atmasital2, Kalajeera4, Nadiaphool, Kalikati2, Basnadhan2, Jawaphool, RamabanaBasmati, Kalkati, Nadiakata, Kalakanhu, Badshahbhog, Gobindabhog, Sitabhog, Krishnabhog, Dubraj, Katrani, Adamchini, Kalanamak-2 | | |
| Hap2 | *O.rufipogon* (KF428978), Leelabati, Kalagiri | | |
| Hap3 | *O.nivara* (AP006728.1), Chatianaki, Atmasital1 | | |
| Hap4 | *O.indica* (93-11), Dhusara, Suman, Magura, Kalazeera, Baluchi, Sunsuniasunaphul, Shantibhog, Phulabanilocal, Basumati3, Tulasibasa, Gopalbhog, Kalanamak-1 | | |
| **Haplotype** | **Name of genotypes** | **Haplotype** | **Name of genotypes** |
| Hap5 | Jayaphul | Hap20 | Nanu |
| Hap6 | Basubhog | Hap21 | Kalaziri |
| Hap7 | Dubraj | Hap22 | Nadiarasa |
| Hap8 | Ganjeikali | Hap23 | Karpurakanta |
| Hap9 | Koiamba543, Thakursuna | Hap24 | Suetpotato |
| Hap10 | Karpurabasa | Hap25 | Basanaparijata |
| Hap11 | Karpurazeera, Benugopal | Hap26 | Deulabhog2 |
| Hap12 | Laxmikajol | Hap27 | Mahulakuchi,Mahulkuchi |
| Hap13 | Karpurakranti | Hap28 | Karpurkali |
| Hap14 | Basasaphool | Hap29 | Basanadhan |
| Hap15 | BhadrakaBasumati | Hap30 | Lektimachi2 |
| Hap16 | Basumati1 | Hap31 | Deulabhog3 |
| Hap17 | Kalakrishna, Basumati2 | Hap32 | Ganjamlocal2 |
| Hap18 | Kalajiri1 | Hap33 | Morllu |
| Hap19 | Laxmibilas2 | - |  |

Hap: haplotype
